# Congenital Anomalies and Variations of the Carotid and Vertebral Arteries: A Case-Based Imaging Review

**DOI:** 10.3390/tomography12080111

**Published:** 2026-07-30

**Authors:** Bilal Battal, Carlos Zamora

**Affiliations:** Division of Neuroradiology, Department of Radiology, University of North Carolina School of Medicine, Chapel Hill, NC 27599, USA; carlos_zamora@med.unc.edu

**Keywords:** neuroimaging, carotid artery, vertebral artery, CTA, MRA, variations, persistent fetal connections, aberrant ICA, persistent trigeminal artery

## Abstract

The carotid and vertebral arteries are critical components of the cerebral circulation, supplying the brain and spinal cord. Their embryologic development involves complex transformations of the aortic arches, paired dorsal aortae, and cervical intersegmental arteries. Congenital anomalies and variations involving their origin, course, and branching patterns are relatively common and are increasingly recognized using modern imaging. While many of these variants are incidental, some carry important clinical implications, particularly in contexts of stroke imaging, head and neck surgery, and neurointerventional procedures. This article reviews the embryologic development of the carotid and vertebral arterial systems, highlights normal and variant anatomy, and presents a case-based, illustrated overview of these congenital anomalies. Emphasis is placed on key findings utilizing computed tomography angiography (CTA), magnetic resonance angiography (MRA), and catheter angiography. Particular attention is given to structural and pathway variations, including vessel agenesis or hypoplasia, the aberrant internal carotid artery within the temporal bone, abnormal branching patterns, fenestrations, and persistent fetal anastomoses (such as the primitive trigeminal, hypoglossal, and proatlantal intersegmental arteries). Ultimately, familiarity with these anomalies, their embryological origins, and their distinct imaging characteristics is essential for ensuring accurate interpretation and avoiding diagnostic pitfalls and procedural complications.

## Simple Summary

Congenital anomalies and variations of the carotid and vertebral arteries are relatively common and are increasingly recognized using modern imaging. While many of these variants are incidental, some carry important clinical implications, particularly in the context of stroke imaging, head and neck surgery, and neurointerventional procedures. This review summarizes the embryologic development of these arterial systems and provides an illustrated overview of normal anatomy and congenital variants using computed tomography angiography, magnetic resonance angiography, and catheter angiography. Familiarity with these anomalies, their embryological origins, and their distinct imaging characteristics is essential for ensuring accurate interpretation and avoiding diagnostic pitfalls and procedural complications.

## 1. Introduction

The carotid and vertebral arterial system constitutes a crucial vascular network supplying blood to the brain and spinal cord. The carotid arteries supply approximately two-thirds of the brain, while the vertebrobasilar system supplies the remaining one-third and contributes to the spinal cord’s blood supply [1,2,3].

In the classic anatomic configuration, the right common carotid artery (CCA) originates from the brachiocephalic artery, while the left arises directly from the aortic arch. Each CCA generally bifurcates at the C3–C4 vertebral level into the internal carotid artery (ICA) and the external carotid artery (ECA). Similarly, the vertebral arteries (VAs) typically originate from the bilateral subclavian arteries, ascend through the neck, and join at the pontomedullary junction to form the basilar artery [4,5].

However, anatomic variants and congenital anomalies involving the formation, origin, course, and branching patterns of these vessels are common. They are increasingly recognized due to the widespread use of computed tomography angiography (CTA) and magnetic resonance angiography (MRA), which provide excellent visualization of vascular structures through high-contrast resolution and multiplanar, three-dimensional (3D) reconstructions [1].

Although these anomalies and variations are often incidental, some have critical implications for stroke imaging, head and neck surgery, and neurointerventional procedures. Recognizing these sometimes subtle imaging appearances and understanding their embryologic origins are essential for accurate detection, avoiding misinterpretation, and preventing procedural complications [1,6,7,8].

The purpose of this review is to provide a brief outline of the embryologic development of the carotid and vertebral arteries, highlight normal and variant anatomy, illustrate congenital anomalies and variations using a case-based approach, and emphasize key findings on CTA, MRA, and catheter angiography.

## 2. Embryology of the Carotid and Vertebral Arterial System

### 2.1. Carotid Arterial System

**Figure 1:**
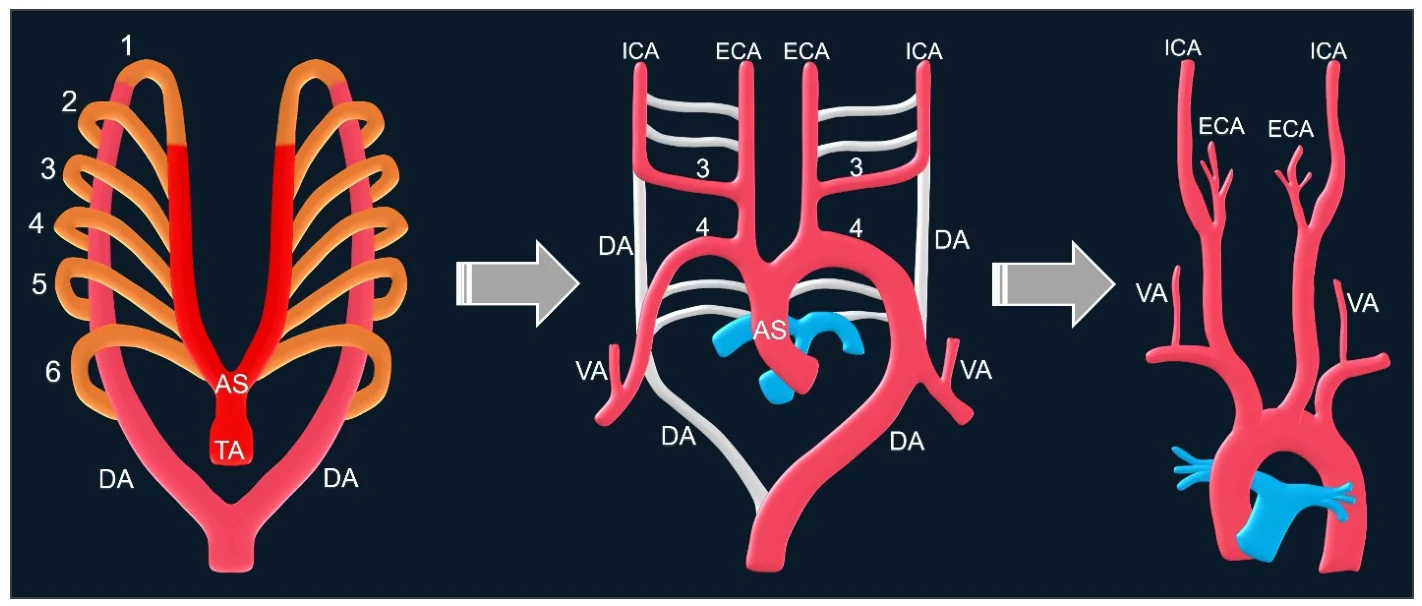
Schematic representation of the establishment of the mature aortic arch, carotid system, and vertebral arteries through a combination of regression (1st, 2nd, part of the 6th aortic arches, and parts of the ventral aortae, AS/TA and DA), persistence, and anastomosis, which is completed by the end of the 6th embryonic week. The 5th aortic arch is rudimentary or frequently absent in humans. 1–6, aortic arches; AS, aortic sac; TA, truncus arteriosus; DA, dorsal aortae; ECA, external carotid artery; ICA, internal carotid artery; VA, vertebral artery.

**Figure 2:**
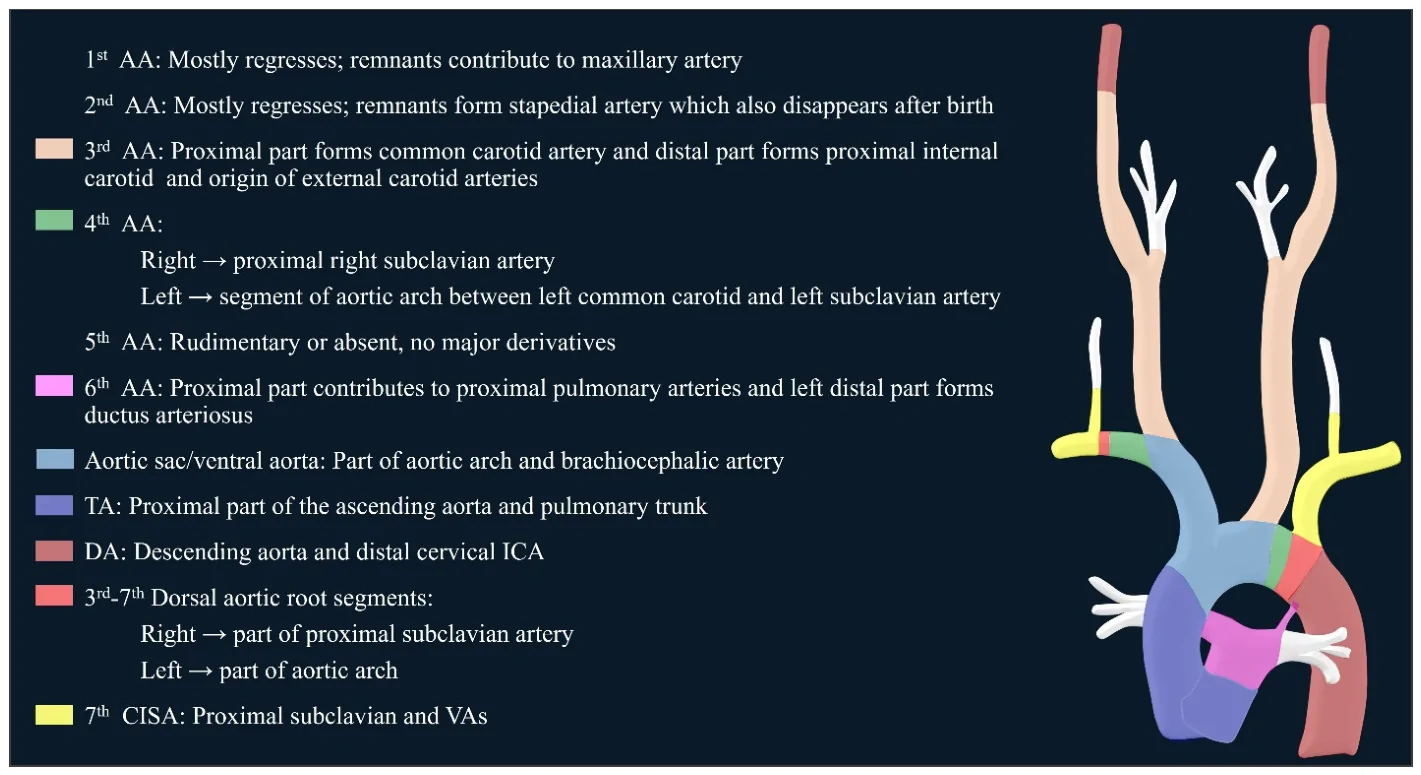
Schematic representation of the structures arising from the truncus arteriosus (TA), aortic sac/ventral aorta, dorsal aortae (DA), aortic arches (AA) and cervical intersegmental arteries. ICA, internal carotid artery; VA, vertebral artery.

The aortic arch and its major branches, including the carotid and vertebral arteries, develop from the aortic sac/ventral aorta, the paired dorsal aortae, the paired aortic arches, and the dorsal intersegmental arteries. During the 4th and 5th weeks of embryonic development, the embryo develops six paired aortic arches that run within the pharyngeal arches along the pharyngeal wall, connecting the truncus arteriosus/aortic sac to the paired dorsal aortae. These six pairs are never present simultaneously; rather, they appear sequentially. Moreover, the fifth aortic arch is generally considered rudimentary or frequently absent in humans. By the time the third pair develops, the first pair has already regressed. The aortic arches then decrease in number and undergo rearrangement. Their selective regression and/or persistence ultimately determine the final great vessel anatomy (Figure 1) [4,6,8,9,10]. Specifically, the brachiocephalic artery is formed by the aortic sac/ventral aorta, while the third aortic arch forms the CCA and the proximal ICA. The dorsal aortae contribute to the formation of the distal ICA. Additionally, each third arch artery gives off a bud that grows cranially to form the ECA [4,8,9,10]. The structures arising from the truncus arteriosus, aortic sac/ventral aorta, dorsal aortae, aortic arches, and dorsal intersegmental arteries are defined and illustrated in Figure 2.

### 2.2. Vertebral Arterial System

**Figure 3:**
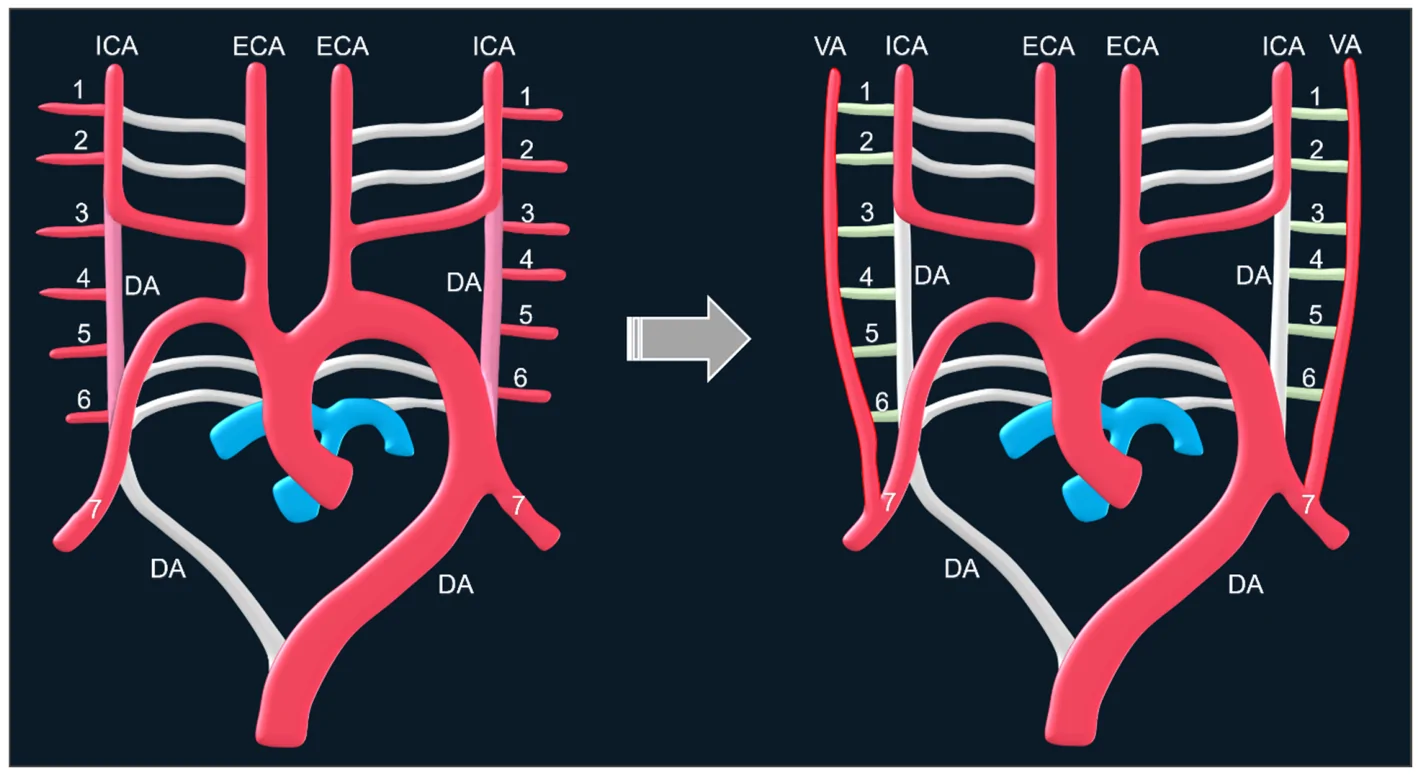
Schematic illustration of vertebral artery development. The paired dorsal aortae give rise to seven pairs of CISAs, corresponding to one per somite in the neck region. The upper six CISAs connect via longitudinal anastomoses to form the precursor VA channel. The proximal transverse connections of the 1st through 6th CISAs to the dorsal aorta regress progressively. The 7th CISA persists. On the right, it combines with the 4th aortic arch to form the proximal subclavian artery. On the left, the 7th CISA forms the proximal left subclavian artery, whereas the left 4th aortic arch contributes to the aortic arch. This establishes the typical origin of the VA from the subclavian artery. 1–7, cervical intersegmental arteries (CISAs); DA, dorsal aortae; ECA, external carotid artery; ICA, internal carotid artery; VA, vertebral artery.

During the 4th week of embryonic development, the paired dorsal aortae give rise to seven pairs of cervical intersegmental arteries (CISAs), corresponding to one per somite in the neck region. The upper six CISAs connect via longitudinal anastomoses to form the precursor channel for the VA. Subsequently, the proximal transverse connections of the 1st through 6th CISAs to the dorsal aorta progressively regress. During the 6th week, the 7th CISA persists. On the right, it combines with the 4th aortic arch to form the proximal subclavian artery. On the left, the 7th CISA forms the left subclavian artery, whereas the left 4th aortic arch contributes to the formation of the aortic arch. This developmental process establishes the typical origin of the VA, which commonly arises from the subclavian artery as its largest and most proximal branch (Figure 3) [4,6,8,9,10]. If one of the first six CISAs fails to regress, the VA may originate anomalously from the ECA, ICA, CCA or the aorta, or may have a dual origin. Specific variations include:

− Persistent 1st or 2nd CISA (horizontal segments): Leads to an aberrant VA origin from the ECA or ICA.
− Persistent 3rd, 4th, or 5th CISA: May result in the VA originating from the right CCA.
− Persistent 6th CISA: Can cause an abnormal VA origin arising from the aortic arch [4,6,8,9,10,11,12].

### 2.3. Fetal Carotid–Vertebral Connections

**Figure 4:**
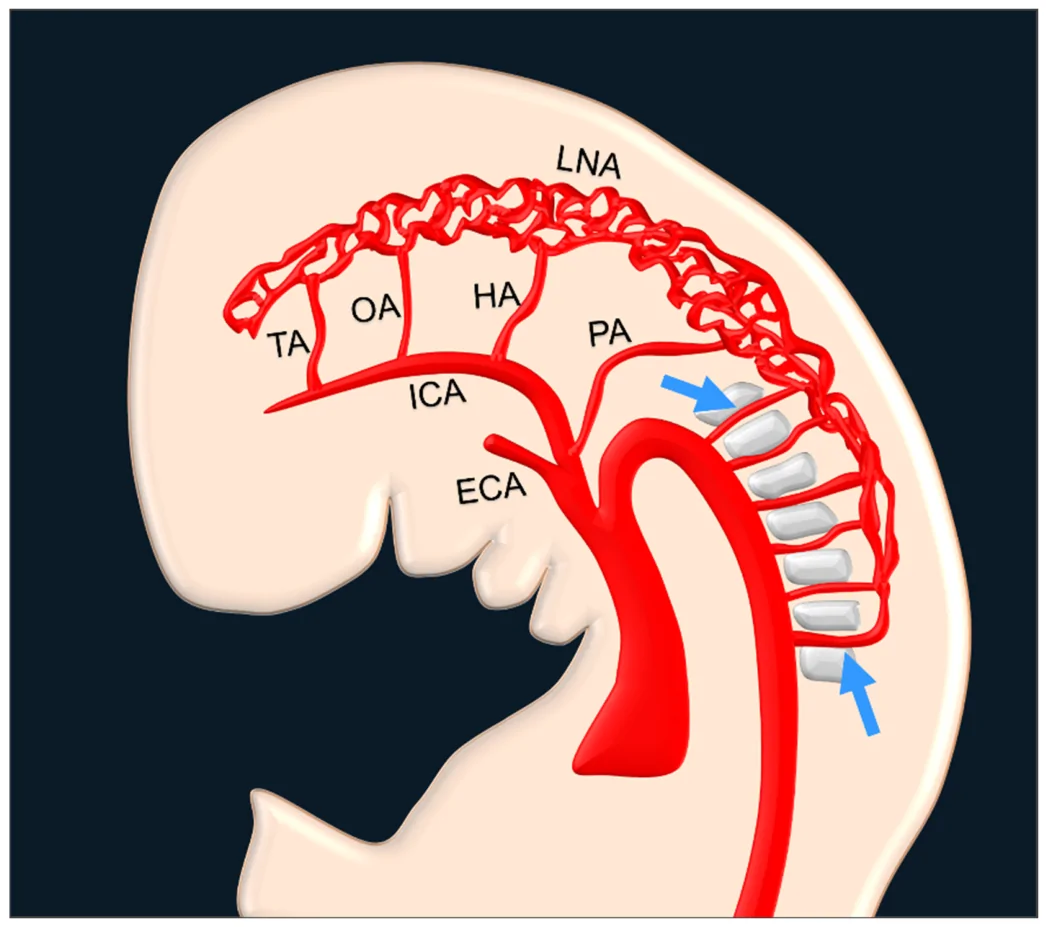
Schematic illustration of the primitive carotid–vertebrobasilar anastomoses, which temporarily provide arterial supply from the internal carotid artery (ICA) to the longitudinal neural artery (LNA). Blue arrows indicate the 1st–6th cervical intersegmental arteries; ECA, external carotid artery; TA, trigeminal artery; OA, otic artery; HA, hypoglossal artery; PA, proatlantal artery.

In the early stages of development, nutrition for the neural tube is provided through diffusion and, subsequently, by a perineural vascular network. During the 5th week of embryonic life, this perineural arterial network establishes communication with the developing cardiac system via the paired aortic arches/carotid system and the longitudinal neural arteries (LNAs) [6,7]. The LNAs are the precursors of the vertebrobasilar system and develop along the ventral wall of the hindbrain. Early embryonic communications link the carotid system to the LNAs through the primitive trigeminal, otic, hypoglossal, and proatlantal arteries (Figure 4) [6,13]. As development progresses, the posterior communicating arteries (PComAs) form, and the VAs arise from the longitudinal anastomoses of the CISAs, which supply the LNAs. The paired LNAs subsequently fuse at the midline to form the basilar artery, completing the vertebrobasilar system.

With the maturation of this system, the primitive carotid–vertebrobasilar connections normally regress. Failure of regression results in variant persistent connections:

− Persistent trigeminal artery (PTA): The most common variant, connecting the cavernous ICA to the basilar artery.
− Persistent hypoglossal artery (PHA): Arises from the cervical ICA and courses through the hypoglossal canal to join the basilar artery.
− Persistent proatlantal artery (PPA): Connects the cervical ICA or ECA to the VA near the foramen magnum.
− Persistent otic artery: Exceedingly rare; its true existence remains uncertain and is still debated in the literature [6,7,14,15].

## 3. Anatomic Considerations

In classic anatomy, the right CCA originates from the brachiocephalic trunk, while the left CCA arises directly from the aortic arch. Each CCA ascends within the carotid sheath in the neck and typically bifurcates at the C3–C4 vertebral level into the ICA and ECA [4].

**Figure 5:**
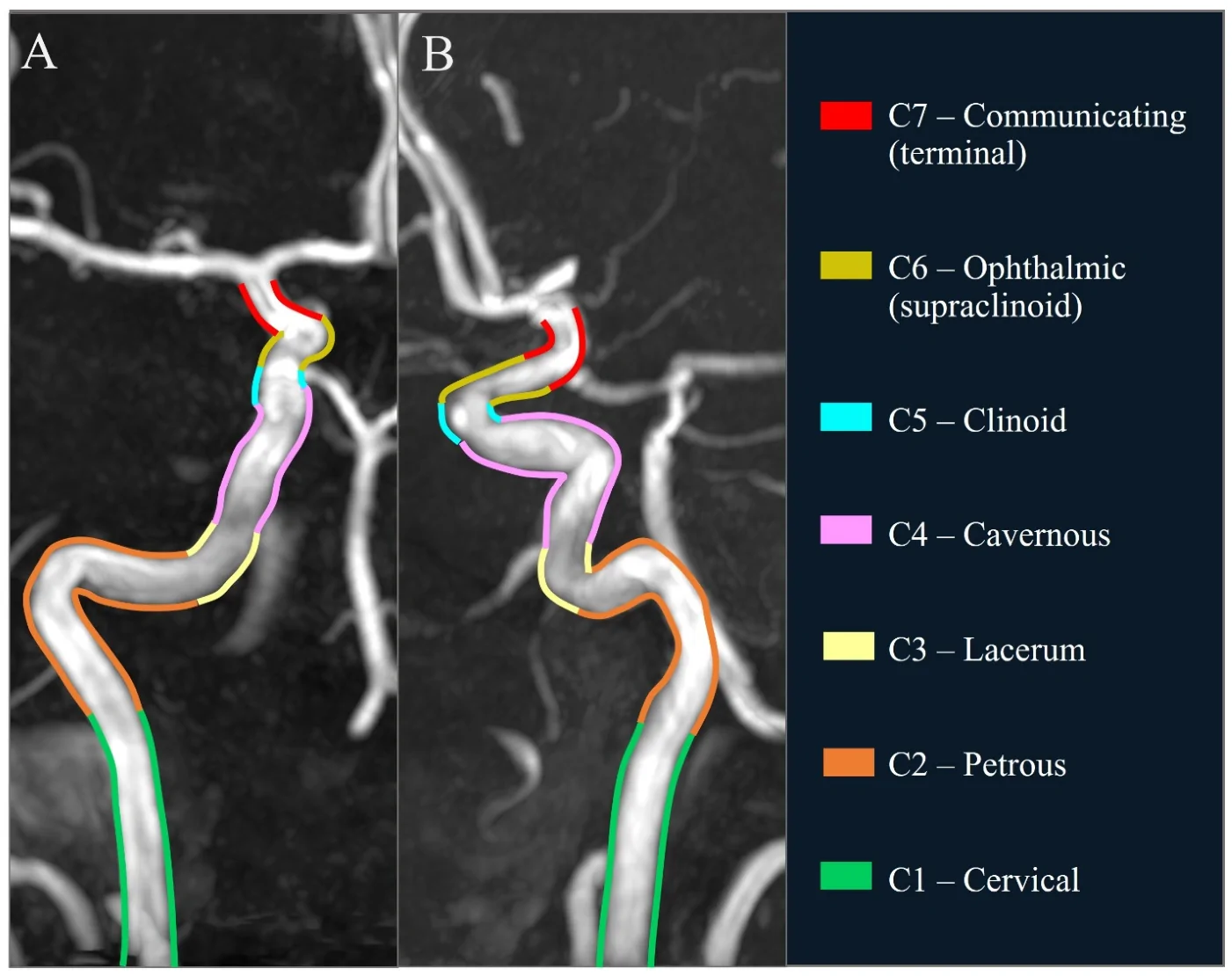
Anteroposterior projection (**A**) and lateral projection (**B**) maximum intensity projection (MIP) MRA images showing the 7 segments of the ICA.

The ECA supplies the face and neck region through multiple extracranial branches, including the superior thyroid, lingual, facial, occipital, maxillary, and superficial temporal arteries. The ICA provides the main arterial supply to the anterior circulation and is classically divided into 7 segments [4,5] (Figure 5):

1. C1—Cervical segment: Extends from the carotid bifurcation to the skull base at the carotid canal. This segment is normally branchless. Although typically straight in younger individuals, it frequently becomes tortuous, coiled, or kinked with advancing age.
2. C2—Petrous segment: Travels within the carotid canal of the temporal bone and gives off the caroticotympanic and vidian arteries.
3. C3—Lacerum segment: A short portion passing over the foramen lacerum without giving off any branches.
4. C4—Cavernous segment: Courses through the cavernous sinus, forming a characteristic S-shaped curvature known as the carotid siphon and gives off the meningohypophyseal trunk.
5. C5—Clinoid segment: A short, branchless transitional portion between the cavernous and intradural segments.
6. C6—Ophthalmic (supraclinoid) segment: Gives off the ophthalmic artery and the superior hypophyseal artery.
7. C7—Communicating (terminal) segment: Gives off the PComA and the anterior choroidal artery. It then bifurcates into the anterior cerebral artery (ACA) and middle cerebral artery (MCA), completing the anterior portion of the circle of Willis (COW).

**Figure 6:**
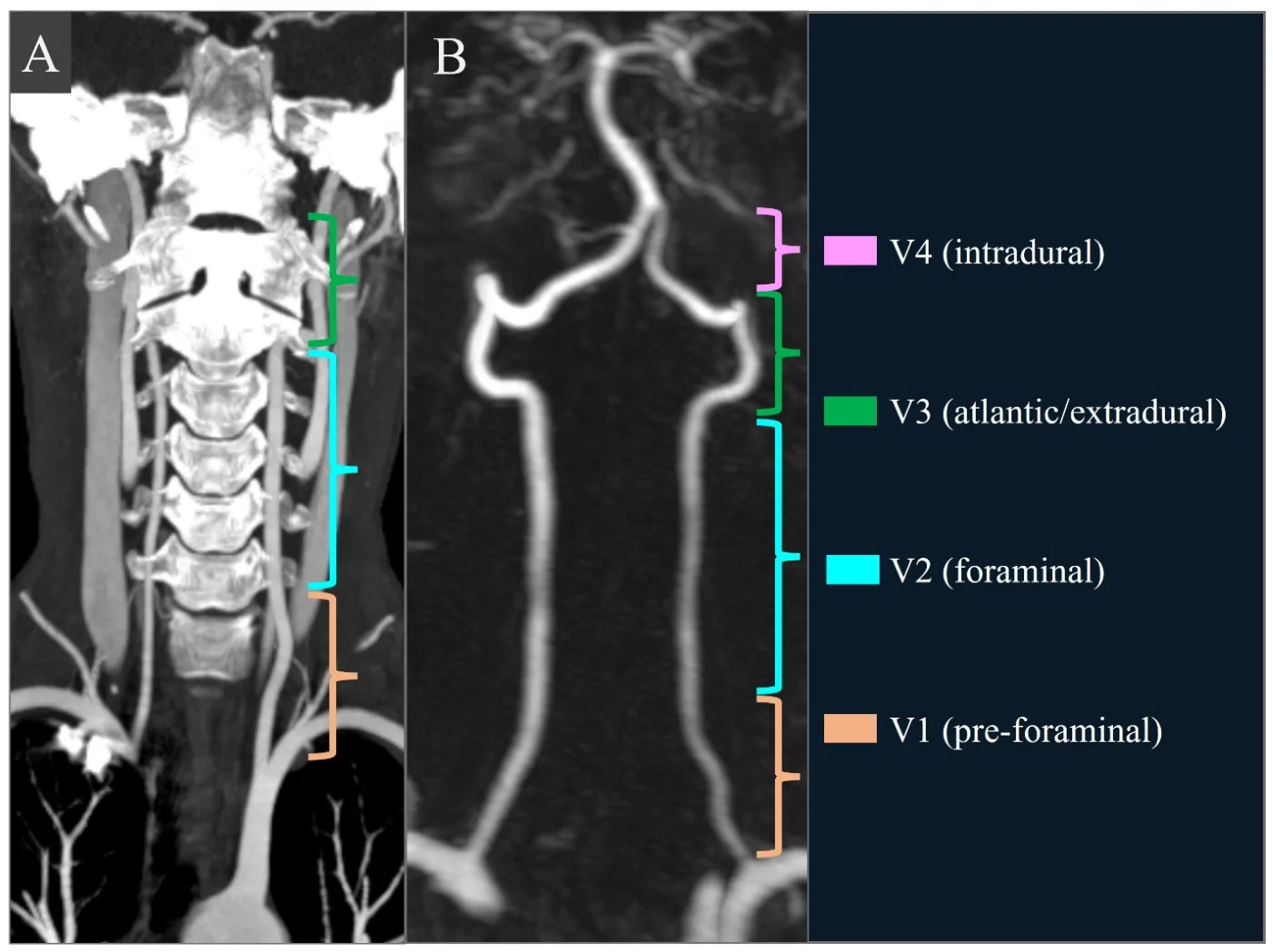
Anteroposterior projection CTA maximum intensity projection (MIP) (**A**) and MRA MIP (**B**) images showing the 4 segments of the VA.

The vertebral arteries usually arise from the subclavian arteries, though origin variations are common. Each VA ascends through the neck and joins its contralateral counterpart at the pontomedullary junction to form the basilar artery, which supplies the posterior circulation. The VAs are divided into four segments (Figure 6):

1. V1 (pre-foraminal) segment: Extends from its origin at the subclavian artery to its entry into the transverse foramen, typically at the C6 level.
2. V2 (foraminal) segment: Ascends through the transverse foramina from C6 to C2.
3. V3 (atlantic/extradural) segment: Exits the transverse foramen of C2, loops laterally and posteriorly around C1, and turns medially to pierce the dura.
4. V4 (intradural) segment: Begins as the artery pierces the dura, courses along the medulla, and joins the contralateral VA to form the basilar artery. This segment generally gives off the posterior inferior cerebellar artery (PICA).

## 4. Imaging Techniques

CTA and MRA are commonly used imaging techniques to evaluate the carotid and vertebral arteries. Although digital subtraction angiography (DSA) is the gold standard—providing superior diagnostic information and enabling therapeutic interventions in selected cases—advances in cross-sectional imaging have significantly decreased the need for invasive diagnostic DSA [1,6,8,11,12,16,17]. Modern multidetector and photon-counting detector (PCD) CT systems can now evaluate very fine vascular structures with shorter scan times and reduced radiation doses. Likewise, high-field MR scanners utilizing volumetric and rapid acquisition sequences offer exceptional vascular detail with shorter acquisition times and without the use of ionizing radiation [1,10,18].

CTA offers high-resolution multiplanar and volumetric 3D images, including maximum intensity projection (MIP) and volume-rendered (VR) reconstructions. These techniques provide excellent anatomical detail regarding the origin, course, developmental anomalies, and variations of the carotid and vertebral arteries, as well as their relationships with surrounding structures. Neck CTA protocols generally require a multidetector CT scanner with at least 64 slices and a thin collimation of 0.5–0.6 mm to obtain isotropic voxels. The imaging field of view should extend from the aortic arch to the cranial vertex, ensuring coverage of the COW. Typically, 60–90 mL of iodinated contrast is administered via an automatic injector at a rate of 4–5 mL/s, followed by a 40 mL saline chaser. In addition to thin axial source images, coronal, sagittal, and curved planar reformations are generated alongside MIP and 3D VR reconstructions. While high spatial resolution, rapid scanning, and wide availability are distinct advantages of CTA, the technique has notable drawbacks. These include risks associated with iodinated contrast, beam-hardening artifacts, and exposure to ionizing radiation, although the latter has been significantly reduced with contemporary multidetector and PCD systems [1,10,18].

MRA techniques include time-of-flight (TOF), contrast-enhanced MRA, and 3D black-blood MRA, typically utilizing 0.5–0.8 mm isotropic voxels. These techniques provide multiplanar and 3D volumetric vessel depiction, as well as crucial information about the vessel wall through vessel wall imaging (VWI), which is useful for evaluating vasculitis, atherosclerosis, and arterial dissection. MRA does not utilize ionizing radiation, and adverse effects of MR contrast agents are relatively rare compared to iodinated contrast. However, longer scan times, susceptibility to motion artifacts, relatively lower spatial resolution compared to CTA, more limited availability, and patient- or device-related contraindications are major drawbacks of this technique [1].

Although its invasive nature and reliance on ionizing radiation limit its diagnostic role, DSA is still regarded as the gold standard for vascular evaluation. Interestingly, congenital anomalies and variations are often more readily identified in their broader anatomical context with noninvasive imaging techniques such as CTA and MRA. Diagnostic DSA requires selective catheterization of the CCA, ICA, ECA, and VAs in multiple projections. Nonionic iodinated contrast, typically 4–8 mL per injection with a flow rate of 4–6 mL/s, is used to acquire angiographic images, usually at a rate of 2–6 frames per second. Three-dimensional rotational angiography is a contemporary technique that is particularly useful and increasingly standard for evaluating aneurysms and complex vascular anatomy. While DSA excels in lumen and dynamic flow assessment, offering unmatched temporal and spatial resolution, its invasive nature, contrast requirements, higher costs, and limited availability outside tertiary centers remain major drawbacks [19].

## 5. Congenital Anomalies and Variations

### 5.1. Congenital Anomalies and Variations of the Carotid Arteries

Congenital anomalies and variations of the carotid arterial system include absence of the ICA, aberrant ICA, high or low bifurcation of the CCA, variant branching patterns, and, in rare cases, duplication and fenestration [4].

#### 5.1.1. Aplasia and Hypoplasia

Congenital absence of the ICA is a rare anomaly with a prevalence of less than 0.01% [4,20,21,22]. It encompasses a spectrum of underdevelopment that is typically unilateral, although rare bilateral cases have been reported:

− Agenesis: Complete failure of the ICA to develop.
− Aplasia: Failure of the ICA to develop fully, leaving behind only rudimentary vestiges.
− Hypoplasia: The presence of an underdeveloped or very small-caliber ICA [23].

The embryologic basis for these conditions involves the failure of development, underdevelopment, or incomplete maturation of the third aortic arch and the cranial segment of the dorsal aorta during early embryogenesis [4].

Clinically, ICA agenesis usually remains asymptomatic and is detected incidentally. This is because collateral blood supply to the brain is maintained through the COW via the anterior communicating artery (AComA) and PComA or, less commonly, through persistent embryonic vessels or transcranial collaterals arising from ECA branches [20,24,25]. However, an absent or hypoplastic ICA can be associated with various conditions, such as neurofibromatosis type 1 (NF-1), NF2-related schwannomatosis, Klippel-Feil syndrome, PHACE syndrome, Goldenhar syndrome, and DiGeorge syndrome [24]. Furthermore, due to altered hemodynamics, these patients have a higher incidence of aneurysm formation in the contralateral carotid system or the COW. Recognizing this anomaly is critical prior to head and neck surgeries (e.g., carotid endarterectomy, carotid ligation, or transsphenoidal surgery) and in the setting of thromboembolic disease.

**Figure 7:**
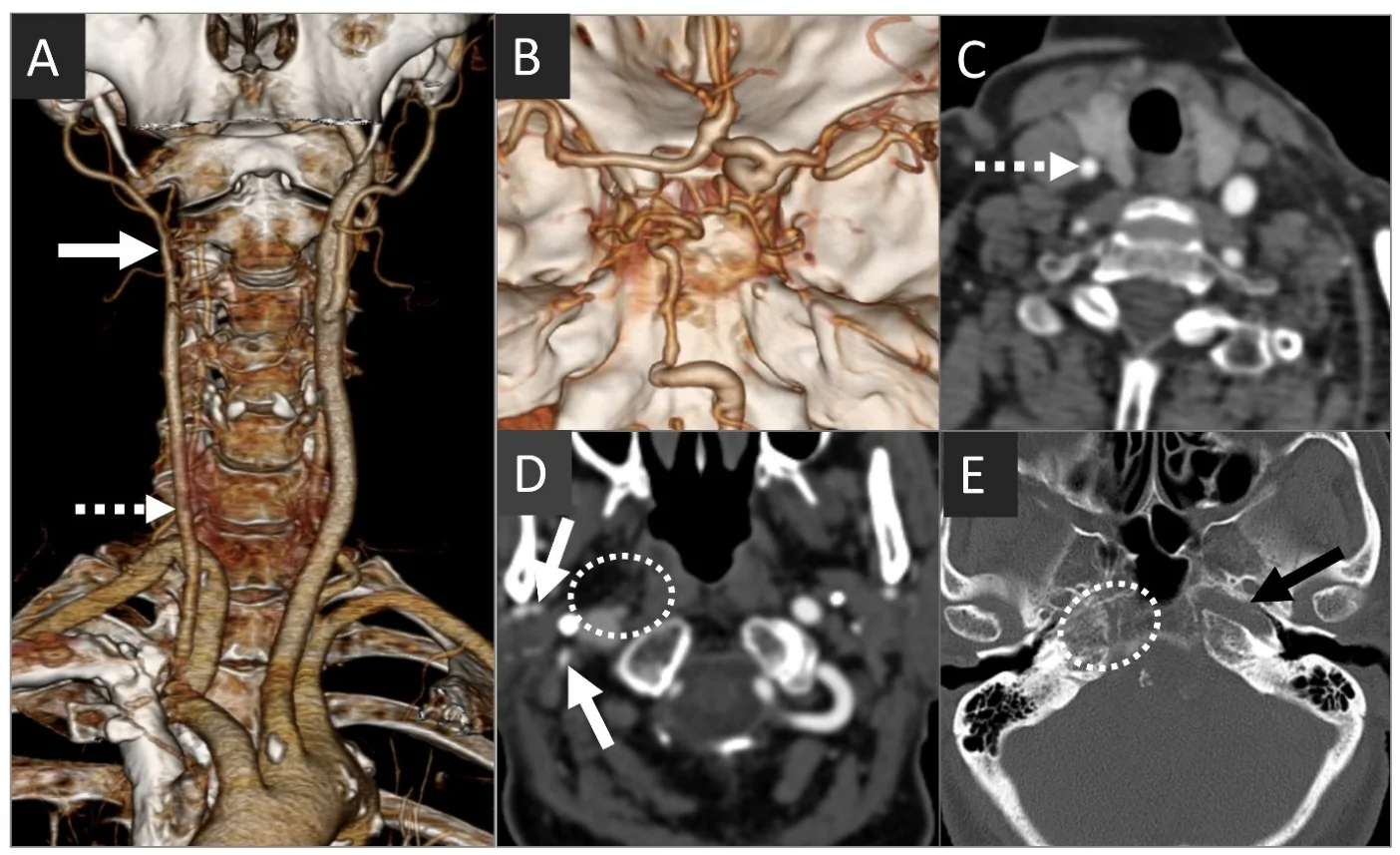
Right ICA agenesis. Volume-rendered (**A**,**B**) and axial (**C**–**E**) CTA images demonstrate an asymmetrically hypoplastic right CCA (dashed arrows) that continues directly as the ECA (white arrows), with an absent right ICA (white circle in (**D**)). The right bony carotid canal is absent (white circle in (**E**)), while the left canal (black arrow) is normal. The right ACA and MCA are supplied by the left ICA via the AComA, and the posterior circulation contributes through the PComA.

**Figure 8:**
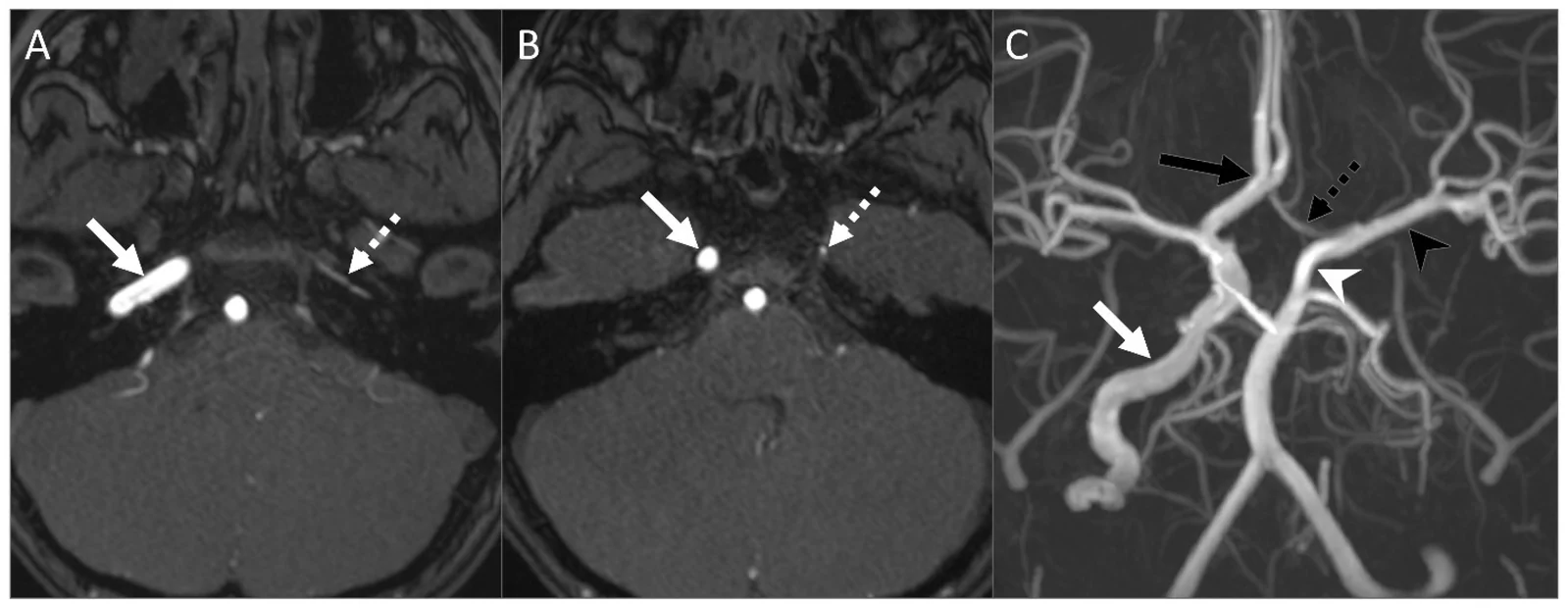
Markedly hypoplastic left ICA. Time-of-flight (TOF) MRA source (**A**,**B**) and MIP (**C**) images demonstrate an asymmetric, markedly hypoplastic left ICA with a narrow carotid canal (dashed white arrows), while the right ICA is normal (white arrows). The left A1 segment of the ACA (dashed black arrow) is hypoplastic; the A2 segment is supplied by the right ICA via the AComA (black arrow), and the left MCA (black arrowhead) is supplied by the posterior circulation via a well-developed left PComA (white arrowhead).

On imaging, agenesis and aplasia demonstrate a complete absence of the ICA along its entire course, whereas hypoplasia presents as a very thin caliber vessel. The key to differentiating congenital absence of the ICA from an acquired occlusion or stenosis lies in the skull base: congenital absence of the ICA is associated with an absent or asymmetrically hypoplastic carotid canal, whereas acquired occlusion or stenosis will have a normal-caliber carotid canal (Figure 7 and Figure 8) [26]. Imaging is also essential for mapping collateral circulation, detecting associated aneurysms, evaluating accompanying syndromic anomalies, and guiding neurovascular interventions.

#### 5.1.2. Aberrant ICA

An aberrant ICA is a rare developmental variant in which the cervical and proximal petrous segments of the ICA fail to form. Instead, embryonic collateral vessels persist to reconstitute the ICA via an abnormal course through the middle ear. In this variant, the inferior tympanic artery (usually a branch of the ascending pharyngeal artery arising from the ECA) enlarges and anastomoses with the caroticotympanic artery (a remnant of the hyoid artery and normally a branch of the ICA).

**Figure 9:**
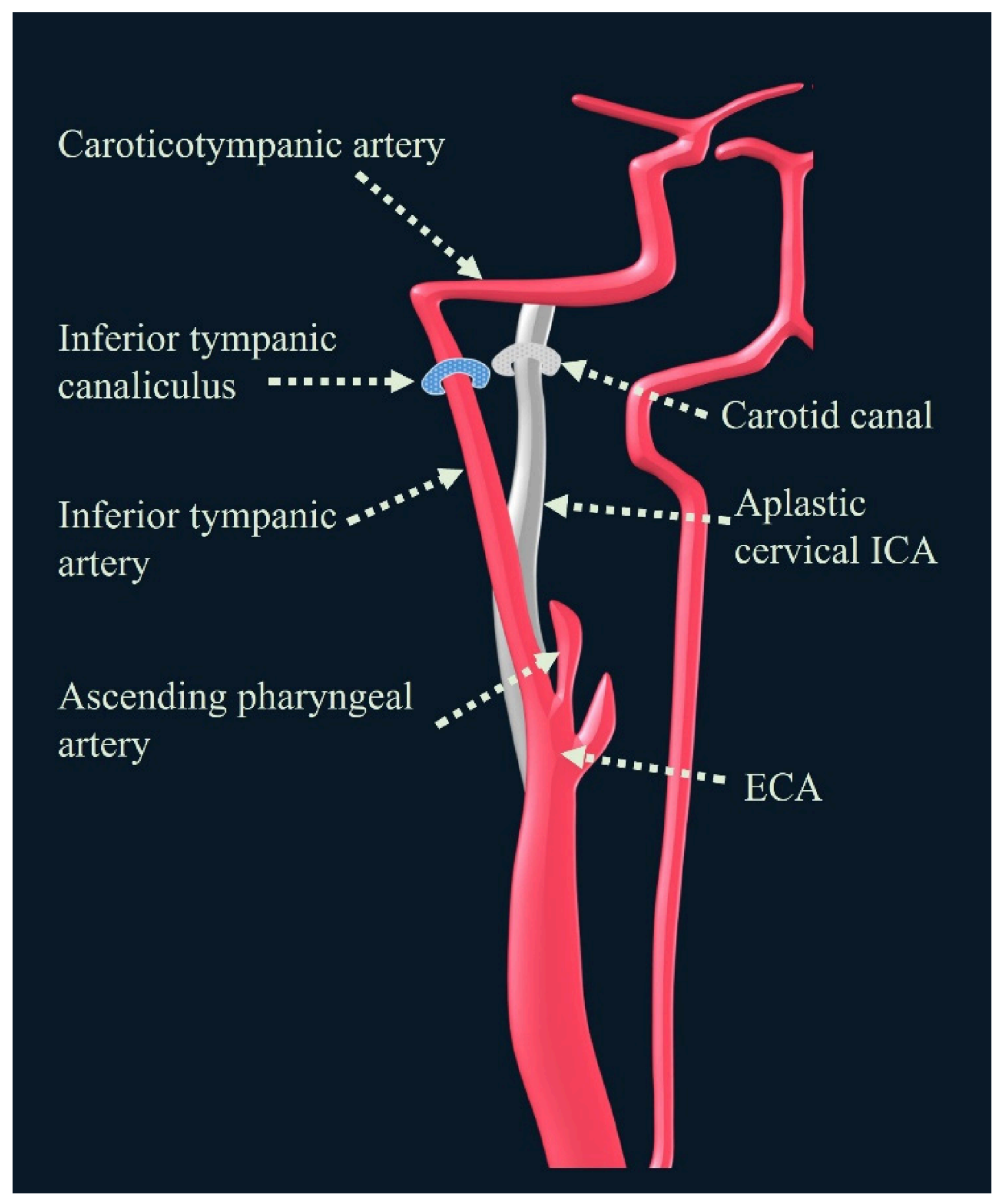
Schematic illustration of an aberrant ICA. This variant results from the developmental failure of the cervical and proximal petrous segments of the ICA, with reconstitution of flow via an enlarged inferior tympanic artery (a branch of the ECA) and the caroticotympanic artery (a branch of the petrous ICA). The aberrant collateral vessels demonstrate a lateralized course and a thin caliber with acute angulation, characteristically resembling a reversed “7” configuration. Additionally, there is enlargement of the inferior tympanic canaliculus, while the normal carotid canal is hypoplastic or absent. ECA, external carotid artery; ICA, internal carotid artery.

This enlarged inferior tympanic artery passes through a widened inferior tympanic canaliculus (also known as the Jacobson’s canal) [27], often demonstrating characteristic narrowing along this segment (Figure 9). The aberrant vessel then enters the posterior mesotympanum, takes a sharp anterior turn, and courses across the inferior cochlear promontory to the anterior mesotympanum. Finally, it joins the normal horizontal portion of the petrous ICA through a dehiscent or absent carotid plate. The normal underlying carotid canal may be hypoplastic or entirely absent.

**Figure 10:**
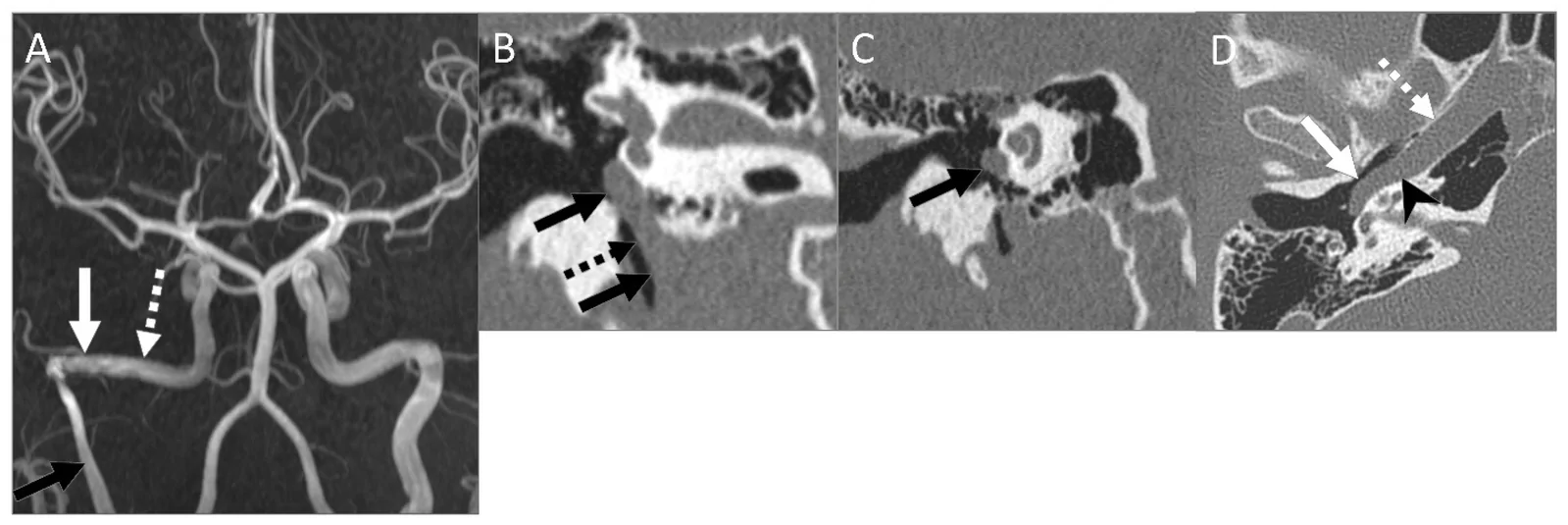
Aberrant right ICA. TOF MRA MIP (**A**) and coronal (**B**,**C**) and axial (**D**) high-resolution computed tomography (HRCT) images of the temporal bone show an enlarged inferior tympanic canaliculus (dashed black arrow). The inferior tympanic artery (black arrows) courses through the middle ear before joining the horizontal petrous ICA (dashed white arrows) via the caroticotympanic artery (white arrow). The aberrant collateral vessels demonstrate a lateralized course and a thin caliber with acute angulation, resembling a reversed number “7” on the coronal plane. The carotid plate is absent (black arrowhead) where the caroticotympanic artery (white arrow) connects to the normal horizontal portion of the petrous ICA (dashed white arrows).

On CTA and MRA, the aberrant ICA demonstrates a reduced caliber and a lateralized course, traversing the enlarged inferior tympanic canaliculus within the caroticojugular spine. On coronal MIP images, the lateralized course and sharp anteromedial angulation in the middle ear resemble the number “7” or a reversed “7” (Figure 10). An aberrant ICA may also be associated with a persistent stapedial artery [27,28].

Clinically, an aberrant ICA is usually asymptomatic but can present with pulsatile tinnitus and hearing loss. Imaging plays a vital role in differentiating this vascular anomaly from middle ear masses (e.g., glomus tympanicum, cholesteatoma, or a high/dehiscent jugular bulb), which may have a similar clinical presentation [29]. Recognition of an aberrant ICA prior to middle ear or skull base surgery is critical, as inadvertent biopsy or injury to the vessel can result in massive hemorrhage, stroke, or death.

#### 5.1.3. Persistent Stapedial Artery (PSA)/Aberrant Carotid Stapedial Artery

A PSA is a rare vascular anomaly with an estimated prevalence of 0.02–0.5% [18,30,31]. It results from the failed regression of the embryonic stapedial artery, a derivative of the second aortic arch.

Normally, the embryonic stapedial artery arises from the hyoid artery (a branch of the dorsal stem of the second arch), passes through the stapes ring, and branches to form the middle meningeal artery (MMA), supplying the maxillofacial region during early development. As the ECA and its branches mature, the stapedial and hyoid arteries regress, leaving behind only small tympanic remnants. Blood supply to the MMA and maxillofacial region is subsequently taken over by the internal maxillary artery (a branch of the ECA).

When the stapedial artery fails to regress, several anatomic variations can occur. These are best categorized proximally by their arterial origins and distally by their transmitting foramina:

Proximal variations:

− Persistent pharyngo-stapedial artery: The PSA arises from the inferior tympanic artery (a branch of the ECA).
− Persistent hyoido-stapedial artery: The PSA arises from the caroticotympanic artery (a branch of the ICA) [18].

Distal variations (at the level of the fallopian canal):

− Duplicated fallopian canal variant: The distal PSA is formed by the superior tympanic artery (a branch of the MMA). It enters the tympanic segment of the fallopian canal through an inferior dehiscence, then exits laterally into a separate bony canal, the superior tympanic canaliculus, just posterior to the cochleariform process. From there, it follows the lesser petrosal nerve.
− Enlarged fallopian canal variant: The distal PSA is formed by the superficial petrosal artery (also a branch of the MMA). In this variant, it courses directly within the fallopian canal alongside the facial nerve.

**Figure 11:**
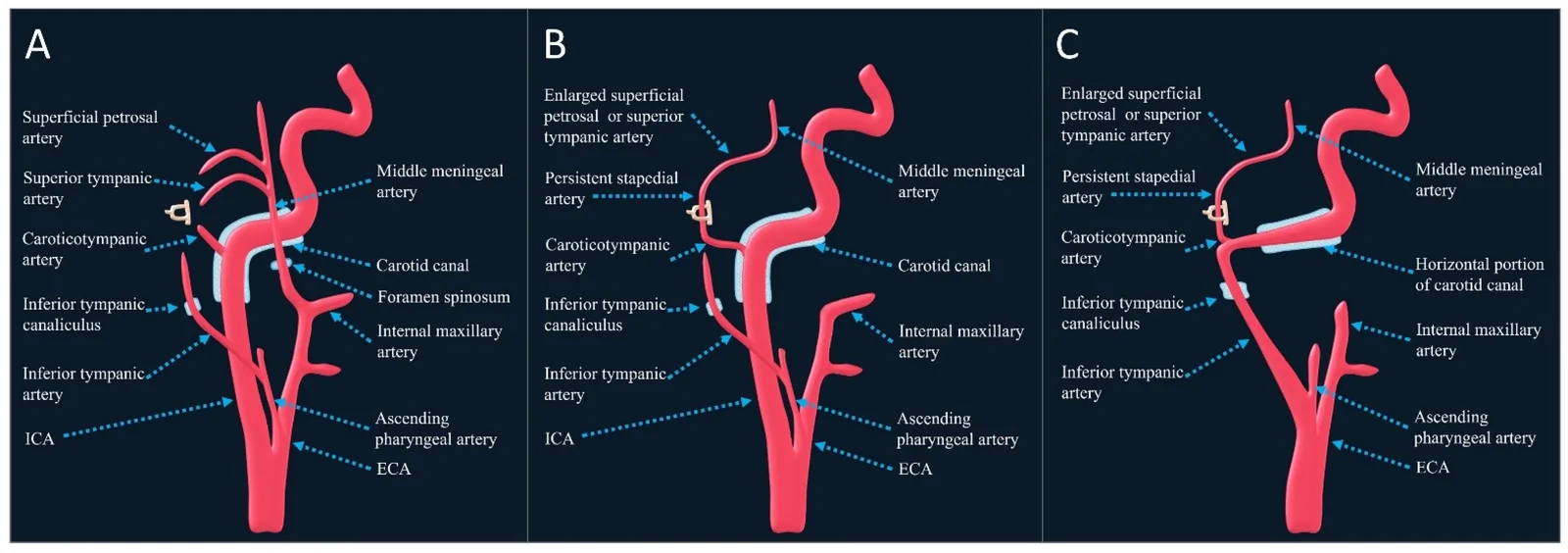
Schematic illustration of the normal anatomy and a portion of the tympanic arterial plexus (**A**), an isolated persistent stapedial artery (**B**), and a persistent stapedial artery associated with an aberrant ICA (termed an aberrant carotid stapedial artery) (**C**). ECA, external carotid artery; ICA, internal carotid artery.

In both distal settings, the PSA subsequently traverses the floor of the middle cranial fossa, giving off the MMA laterally and anastomotic branches to the orbital vessels medially (Figure 11).

**Figure 12:**
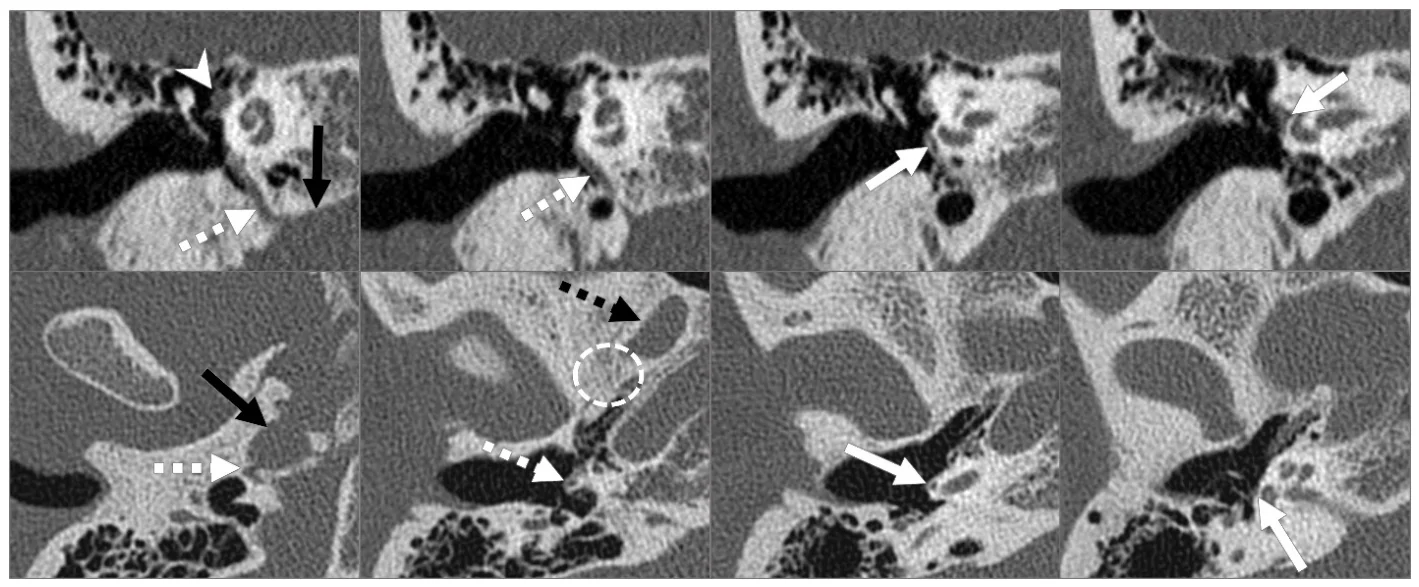
Persistent stapedial artery. Coronal (upper row) and axial (lower row) HRCT images of the temporal bone demonstrate the absence of the foramen spinosum (white circle) posterior to the foramen ovale (dashed black arrow), indicating that the middle meningeal artery arises from the ICA instead of the ECA. A small vascular canal (dashed white arrows) arising from the petrous ICA (black arrows), corresponding to the caroticotympanic artery canal, extends superiorly along the cochlear promontory and the stapes footplate, consistent with a persistent stapedial artery (white arrows). There is enlargement of the anterior aspect of the fallopian canal, suggesting a persistent stapedial artery distally formed by the superficial petrosal artery (white arrowhead).

Radiologically, almost all types of PSA, except for the complete hyoido-stapedial artery variant, are associated with the ipsilateral absence of the foramen spinosum. However, this finding is not specific to PSA, as it may also be seen with other variant origins of the MMA [18,32]. On cross-sectional imaging, a PSA is identified by an absent foramen spinosum coupled with a small vascular channel arising from the petrous ICA (persistent hyoido-stapedial artery) or inferior tympanic artery (persistent pharyngo-stapedial artery). This channel extends superiorly along the cochlear promontory, passes through the stapes ring/footplate, and enters the fallopian canal, resulting in the enlargement or duplication of its anterior portion (Figure 12) [18].

Clinically, a PSA is usually asymptomatic and detected incidentally, although it may be associated with pulsatile tinnitus and conductive hearing loss [18]. Preoperative recognition of a PSA is crucial to prevent severe hemorrhage during middle ear and mastoid surgeries. In selected symptomatic cases, surgical transection of the vessel to resolve pulsatile tinnitus and/or ossicular chain reconstruction for conductive hearing loss may be performed [18,31,33,34].

Notably, a PSA may coexist with an aberrant ICA, as both anomalies reflect the persistence of embryonic carotid-tympanic collateral pathways. This exceedingly rare combination is termed an aberrant carotid stapedial artery (Figure 11) [18,35].

#### 5.1.4. Variant Branching of the CCA and Cervical ICA

**Figure 13:**
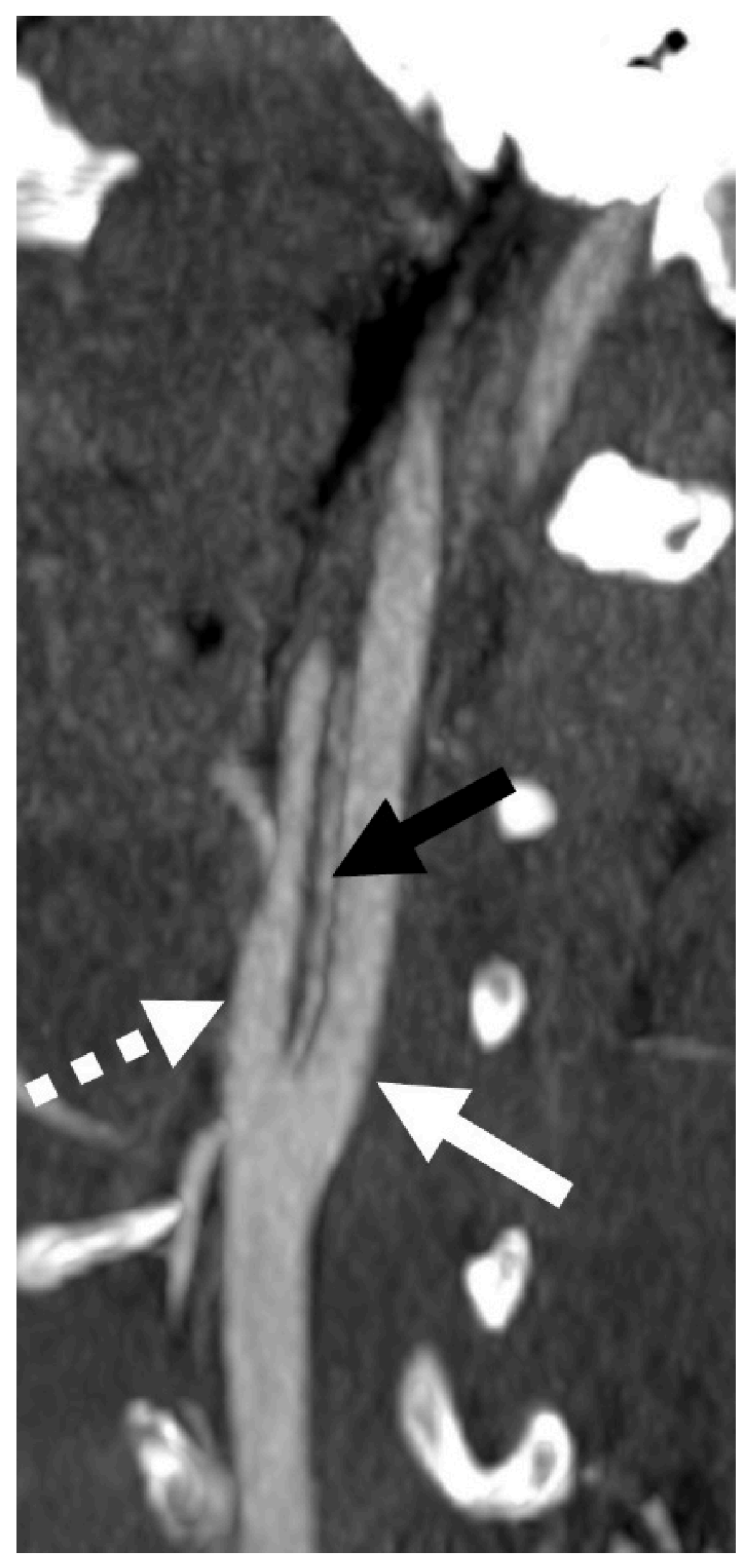
Carotid trifurcation. Sagittal MIP CTA shows carotid trifurcation into the ECA (dashed white arrow), ICA (white arrow), and ascending pharyngeal artery (black arrow).

**Figure 14:**
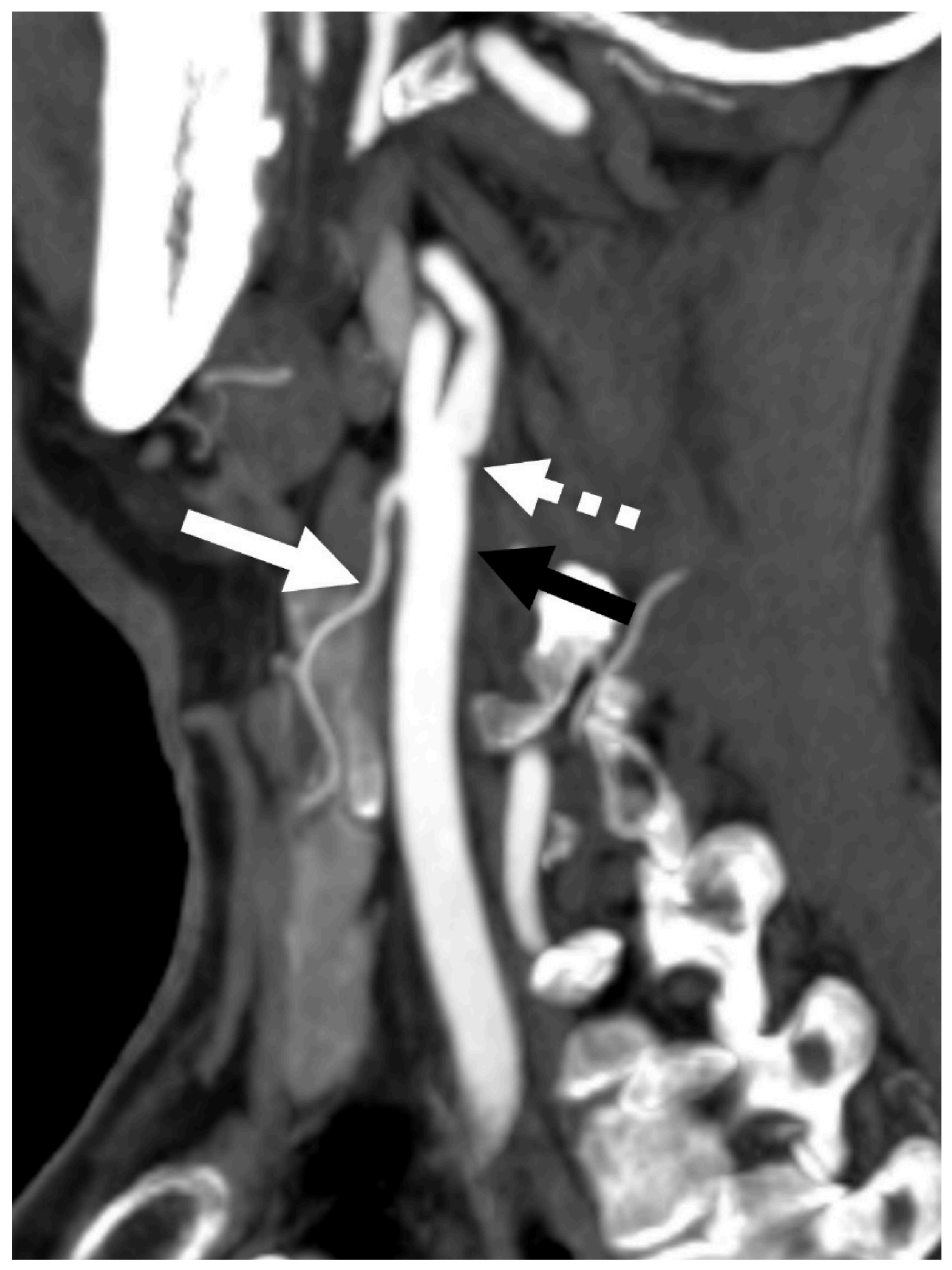
Superior thyroid artery originating from the CCA. Sagittal MIP CTA image shows the left superior thyroid artery (white arrow) arising from the CCA (black arrow) just proximal to the carotid bifurcation. An incidental carotid web is noted (dashed white arrow).

**Figure 15:**
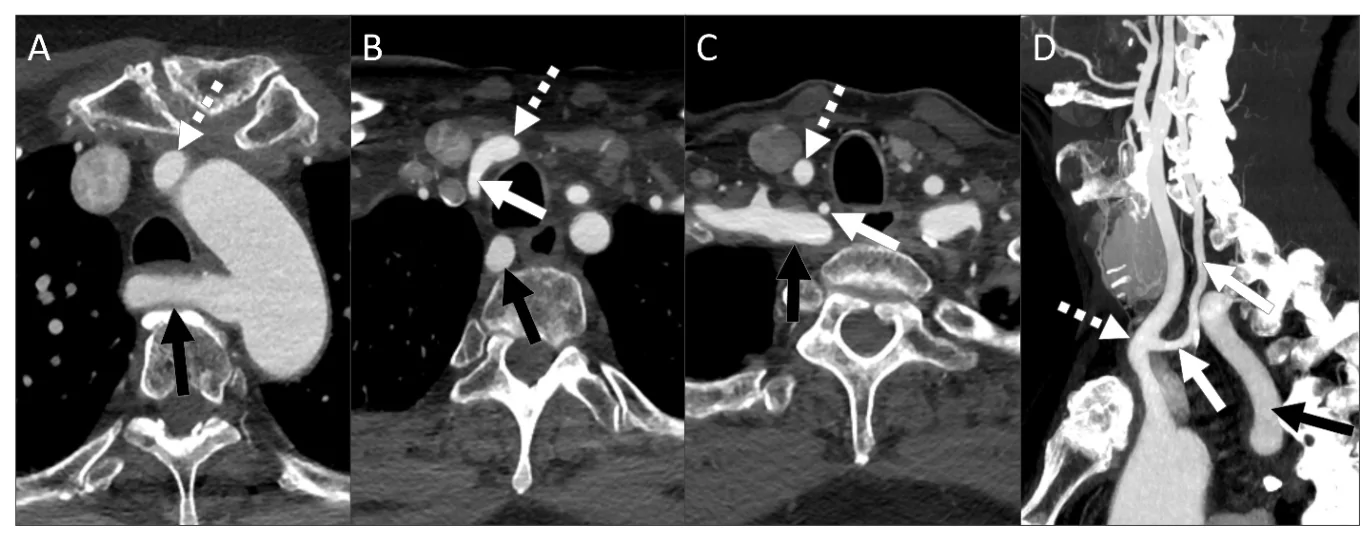
Right VA originating from the right CCA. Axial (**A**–**C**) and oblique sagittal MIP (**D**) CTA images demonstrate an aberrant right subclavian artery (black arrows) originating from the distal aortic arch and an aberrant origin of the right VA (white arrows) from the proximal right CCA (dashed white arrows).

The CCA typically bifurcates at the level of the C3–C4 vertebrae, although a high or low bifurcation may occur, ranging anywhere from the C1 to T2 vertebral levels. Trifurcation is a rare CCA branching variant in which the CCA divides into the ICA, ECA, and an additional branch, most commonly the superior thyroid artery or the ascending pharyngeal artery (Figure 13) [36,37]. Agenesis of the CCA is an extremely rare developmental anomaly characterized by separate origins of the ICA and ECA. In this setting, these vessels arise directly from the brachiocephalic and subclavian arteries on the right, and directly from the aortic arch on the left [38,39]. Occasionally, some ECA branches, such as the superior thyroid, lingual, or facial arteries, and, exceedingly rarely, the vertebral artery, may originate directly from the CCA (Figure 14 and Figure 15) [16,36,37].

**Figure 16:**
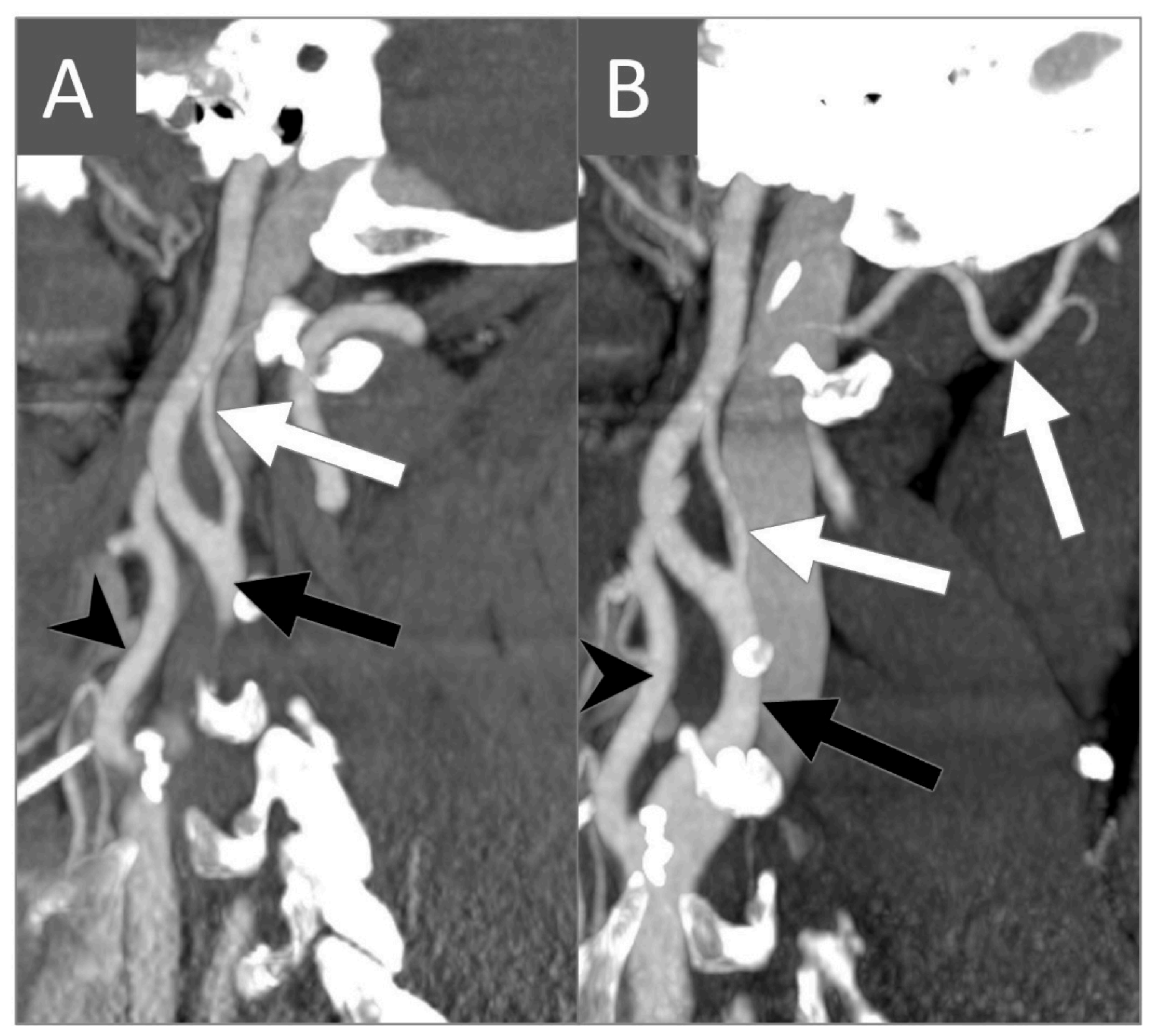
Occipital artery originating from the left ICA. Sagittal CTA MIP images (**A**,**B**) show an aberrant origin of the occipital artery (white arrows) from the ICA (black arrows). Black arrowhead: ECA.

Variant branching of the cervical ICA is uncommon, as this segment normally does not give off branches. In very rare instances, the ascending pharyngeal artery or the occipital artery may arise aberrantly from the cervical ICA (Figure 16). These aberrant carotid artery branching patterns likely reflect persistent embryonic connections between the dorsal aorta, which is the precursor of the ICA, and the primitive branchial arteries. Such variations of the carotid arterial system in the neck may alter the normal imaging appearance of the carotid space vasculature. Recognizing these variant branching patterns is crucial, as they have important implications during head and neck surgeries and endovascular procedures [36].

### 5.2. Congenital Anomalies and Variations of the VAs

#### 5.2.1. Aplasia and Hypoplasia

Congenital absence (aplasia) of the VA is a rare developmental variant resulting from failure of the longitudinal anastomoses to form between the CISAs during early embryogenesis [40]. In contrast, incomplete development or insufficient reinforcement between the CISAs results in VA hypoplasia, a condition in which the VA is diminutive in caliber but remains patent [4,40]. Although definitions vary among studies, a VA diameter less than 2 mm, or a diameter more than 50% smaller than the contralateral VA, is generally considered hypoplastic [1,4,40].

Clinically, VA hypoplasia is a relatively common variation that is frequently identified incidentally on imaging studies. It is usually asymptomatic due to adequate compensation by the contralateral VA and collateral circulation through the COW. However, increasing evidence suggests that VA hypoplasia may predispose patients to posterior circulation ischemia, particularly when the contralateral VA is diseased or additional vascular risk factors are present. Several studies and meta-analyses have suggested a possible association between VA hypoplasia and an increased frequency of posterior circulation stroke. Proposed mechanisms include reduced blood flow, regional hypoperfusion, and increased susceptibility to atherosclerotic and thrombotic changes within the dominant VA [41].

**Figure 17:**
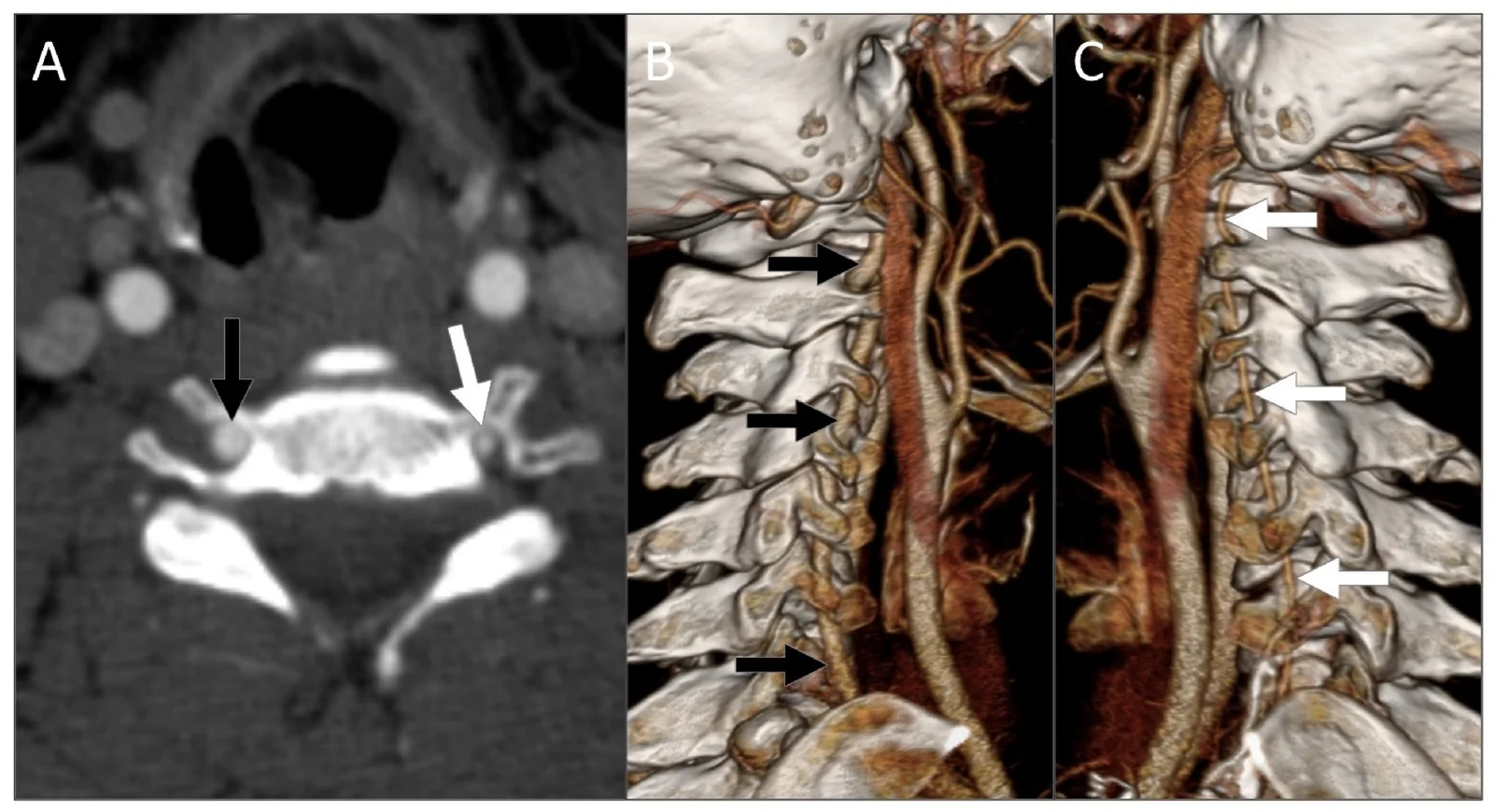
Hypoplastic left VA. Axial CTA (**A**) and volume-rendered (**B**,**C**) images demonstrate an asymmetric, hypoplastic left VA (white arrows) with a dominant right VA (black arrows). The left-sided transverse foramina are also congenitally hypoplastic.

Radiologically, aplasia demonstrates a complete absence of the VA. In these cases, the ipsilateral transverse foramina may be asymmetrically small or absent entirely. This osseous finding supports a congenital etiology, as the transverse foramina develop in conjunction with the adjacent VA. A hypoplastic VA appears uniformly diminutive in caliber but remains patent, often presenting with associated smaller transverse foramina (Figure 17). Additionally, a hypoplastic VA may terminate as the PICA without joining the basilar artery. In contrast, the presence of normal-sized transverse foramina combined with absent VA flow or irregular vessel narrowing is highly suggestive of acquired vascular disease, such as atherosclerosis, vasculitis, dissection, or occlusion. Finally, aplasia and severe hypoplasia of the VA may coexist with persistent embryonic carotid–vertebrobasilar anastomoses, including persistent trigeminal, hypoglossal, and proatlantal arteries [6,40].

#### 5.2.2. Origin and Course Variations of the VAs

Typically, the VA is the first branch arising from the ipsilateral subclavian artery and enters the transverse foramen at the C6 vertebral level. However, various anomalies regarding its origin, transverse foramen entry level, and anatomical course have been reported. These variations result from the abnormal persistence or regression of different embryonic CISAs [6,11,12].

**Figure 18:**
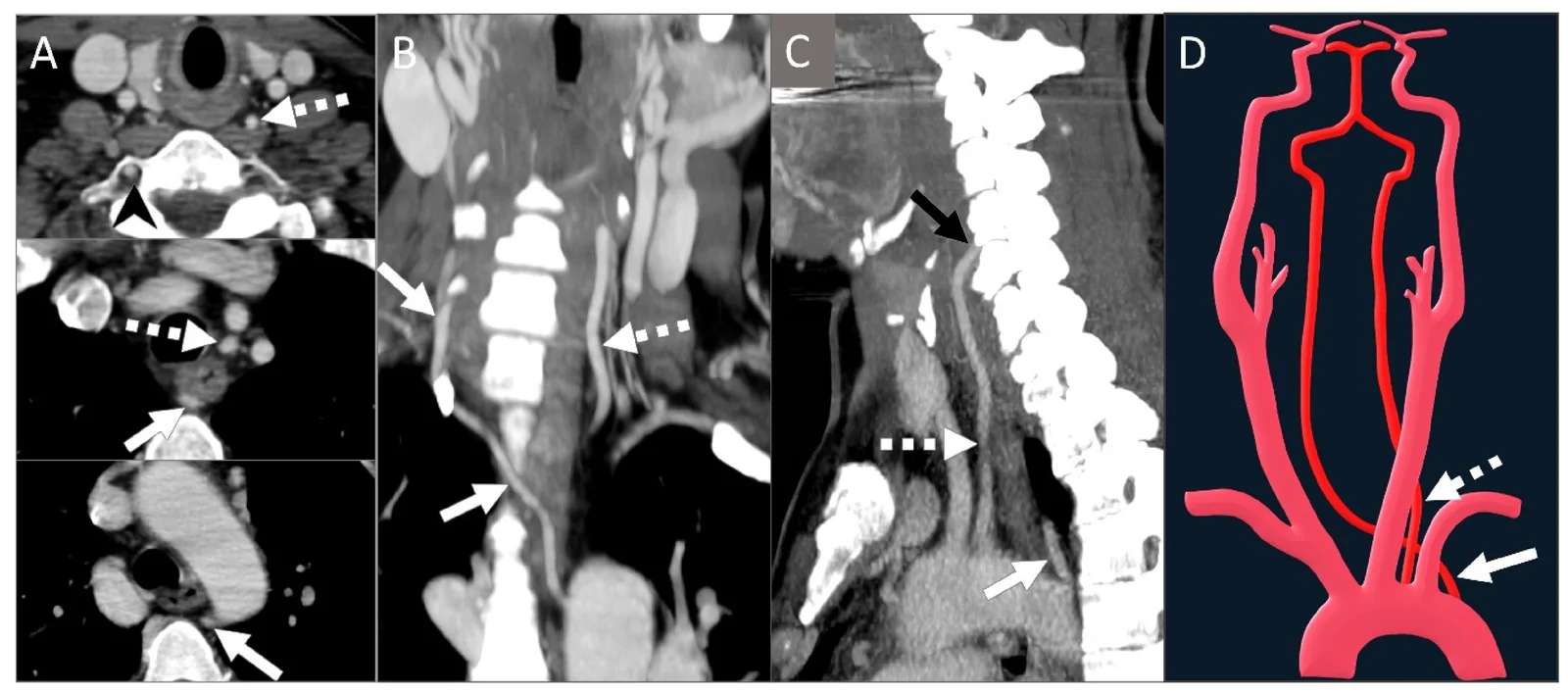
Origin and course variations of the bilateral vertebral arteries. Axial CT (**A**) and oblique coronal and sagittal MIP (**B**,**C**) images, alongside an illustration (**D**), demonstrate an aberrant origin of the right VA (white arrows) from the aortic arch, distal to the origin of the left subclavian artery, coursing posterior to the esophagus and entering the transverse foramen at C7 (black arrowhead in (**A**)). The left VA (dashed white arrows) originates directly from the aortic arch between the left CCA and the left subclavian artery, entering the transverse foramen at C5 (black arrow in (**C**)).

Origin anomalies:

− Aortic arch origin of the left VA: This is the most common origin anomaly, seen in 2–6% of the population [6,42,43,44]. It reflects the persistence of the 6th rather than the 7th CISA. In this variant, the left VA typically arises directly from the aortic arch between the left CCA and left subclavian artery.
− Aberrant aortic arch origin of the right VA: This is much rarer than its left-sided counterpart. The right VA may originate from the aortic arch between the brachiocephalic trunk and the left CCA. Extremely rarely, due to persistence of the right dorsal aorta, it may arise distal to the left subclavian artery (Figure 18) [11,12].
− Other rare origins: The VA may occasionally arise from the CCA, the thyrocervical trunk, or the costocervical trunk due to the anomalous fusion of the CISAs [6,12,16].

**Figure 19:**
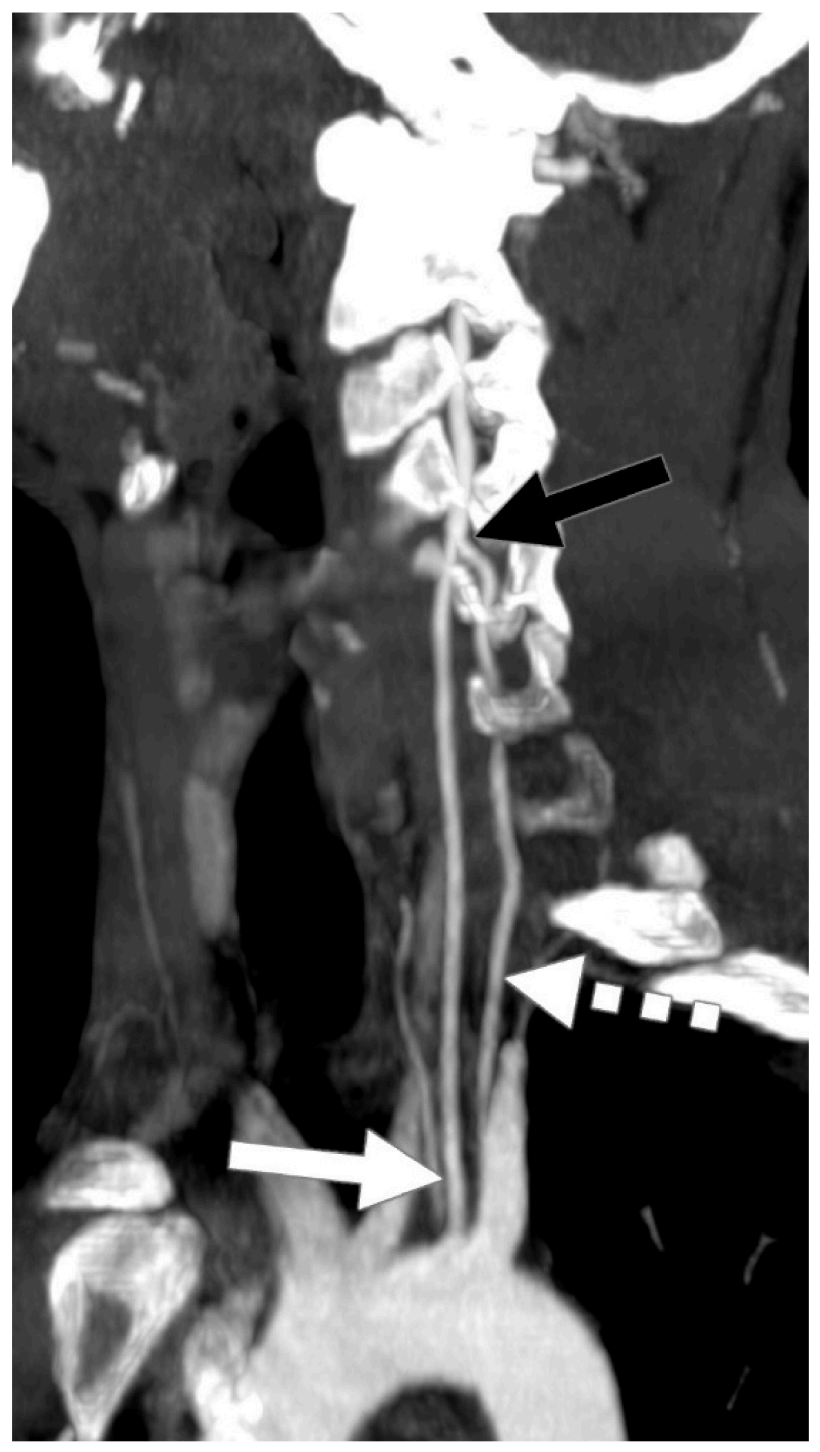
Dual origin of the left VA. Oblique sagittal CTA MIP shows the dual origin of the left VA from the aortic arch (white arrow) and the left subclavian artery (dashed white arrow), merging at the C4–C5 level (black arrow). The aortic arch branch enters the transverse foramen after the confluence.

**Figure 20:**
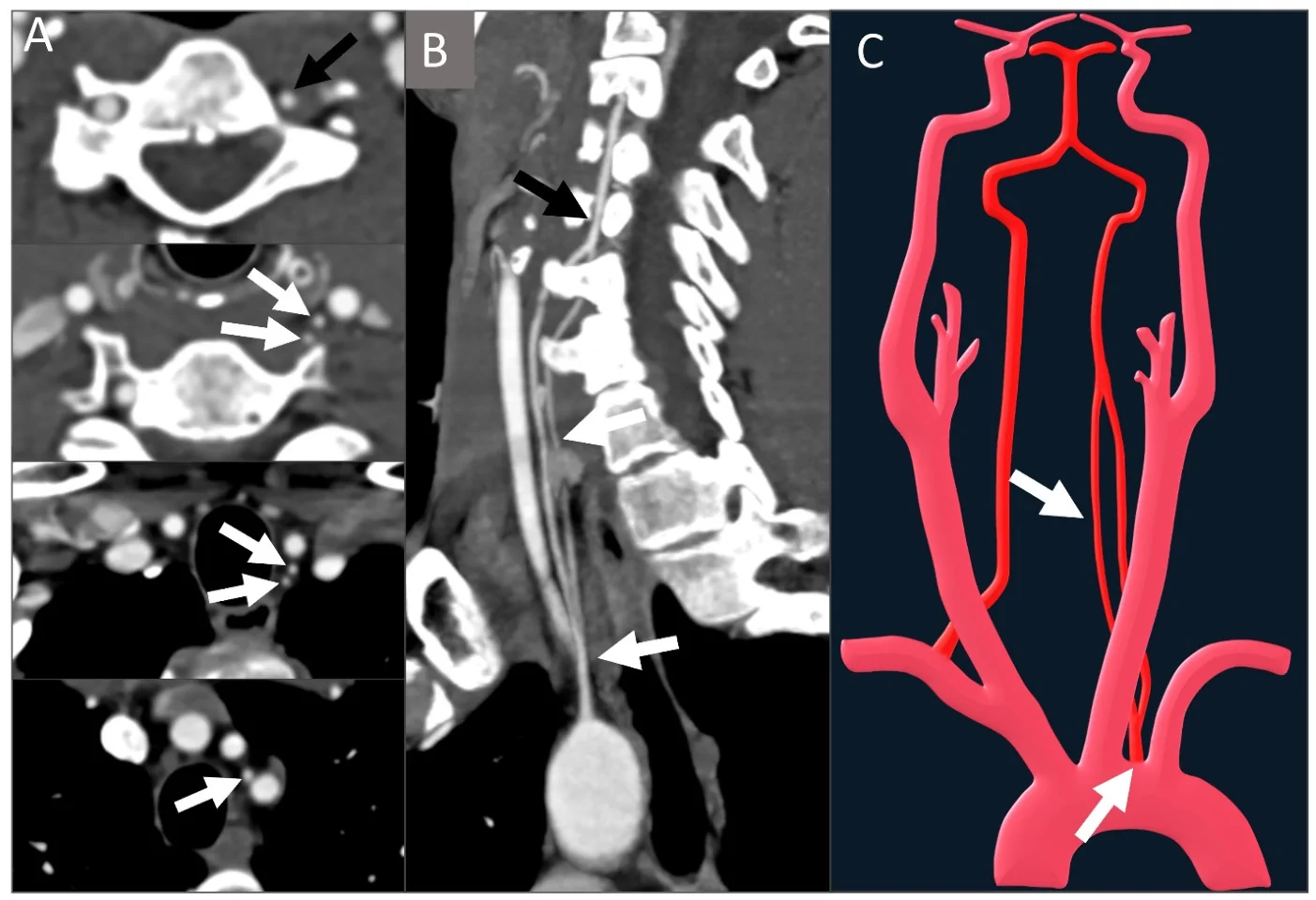
Long-segment fenestration of the left VA originating from the aortic arch. Axial (**A**) and oblique curved sagittal (**B**) post-contrast CTA images, alongside an illustration (**C**), demonstrate a long-segment fenestration of the left VA (white arrows), which originates from the aortic arch between the left CCA and the left subclavian artery, entering the transverse foramen at the level of C4 (black arrows).

Dual origin vs. fenestration:

− Dual origin: Occurs when more than one embryologic precursor vessel persists proximally, causing two distinct limbs to unite at a higher cervical level. Generally, one limb originates from the normal anatomical location (the proximal subclavian artery) while the other arises from an anomalous origin. Rarely, both limbs may have anomalous origins (Figure 19) [6,42].
− Fenestration: Occurs in approximately 0.2–2% of individuals and may affect any segment of the VA [45,46]. Fenestration is a distinct entity from dual origin: whereas duplication involves two separate vessel origins that fuse together, fenestration involves a single artery that splits and subsequently re-fuses (Figure 20) [47,48]. Fenestration of the V2 and V3 segments results from the incomplete fusion of the embryonic longitudinal channels [6,46]. It is usually an incidental finding but is clinically significant because it can be easily mistaken for a vascular dissection or focal stenosis on imaging studies.

Transverse foramen entry levels:

Variant transverse foramen entry levels of the VA are relatively common. The embryologic basis for these variations lies in the selective regression and persistence of the CISAs and their longitudinal connections. The final entry level is determined by which embryonic segmental artery remains functional to supply the longitudinal anastomotic chain that becomes the adult VA [6].

Entry at C5 is reported in 6.5% of cases on the left, while C4 entry is reported in 2.2% on the right [6,42,43]. Entry at C3 or higher may occur but is rare [49]. Conversely, C7 level entry is the rarest variant and frequently accompanies an aortic arch origin of the corresponding VA [6].

### 5.3. Persistent Fetal Carotid–Vertebrobasilar Connections

The primitive trigeminal, otic, hypoglossal, and proatlantal arteries are fetal anastomoses that emerge during early embryonic life. They persist for approximately one week and regress at roughly the same rate as the development of PComAs and VAs. The otic artery is the rarest and the first to disappear, followed by the hypoglossal, trigeminal, and proatlantal arteries [7,15]. A persistent otic artery is exceedingly rare, and its true existence remains uncertain and is still debated in the literature [15].

#### 5.3.1. Persistent Trigeminal Artery (PTA)

The PTA is the most common carotid-basilar anastomosis and is seen in approximately 0.1–0.6% of healthy individuals [7,50]. It results from the failure of regression of the primitive trigeminal artery, which normally connects the cavernous ICA to the basilar artery during early embryogenesis. Various classification systems for the PTA have been proposed based on its origin from the ICA, its anatomical course, and its connection with the posterior circulation (including the Saltzman, Salas, and Weon classifications), although some variants show overlapping or hybrid features [51,52,53].

**Figure 21:**
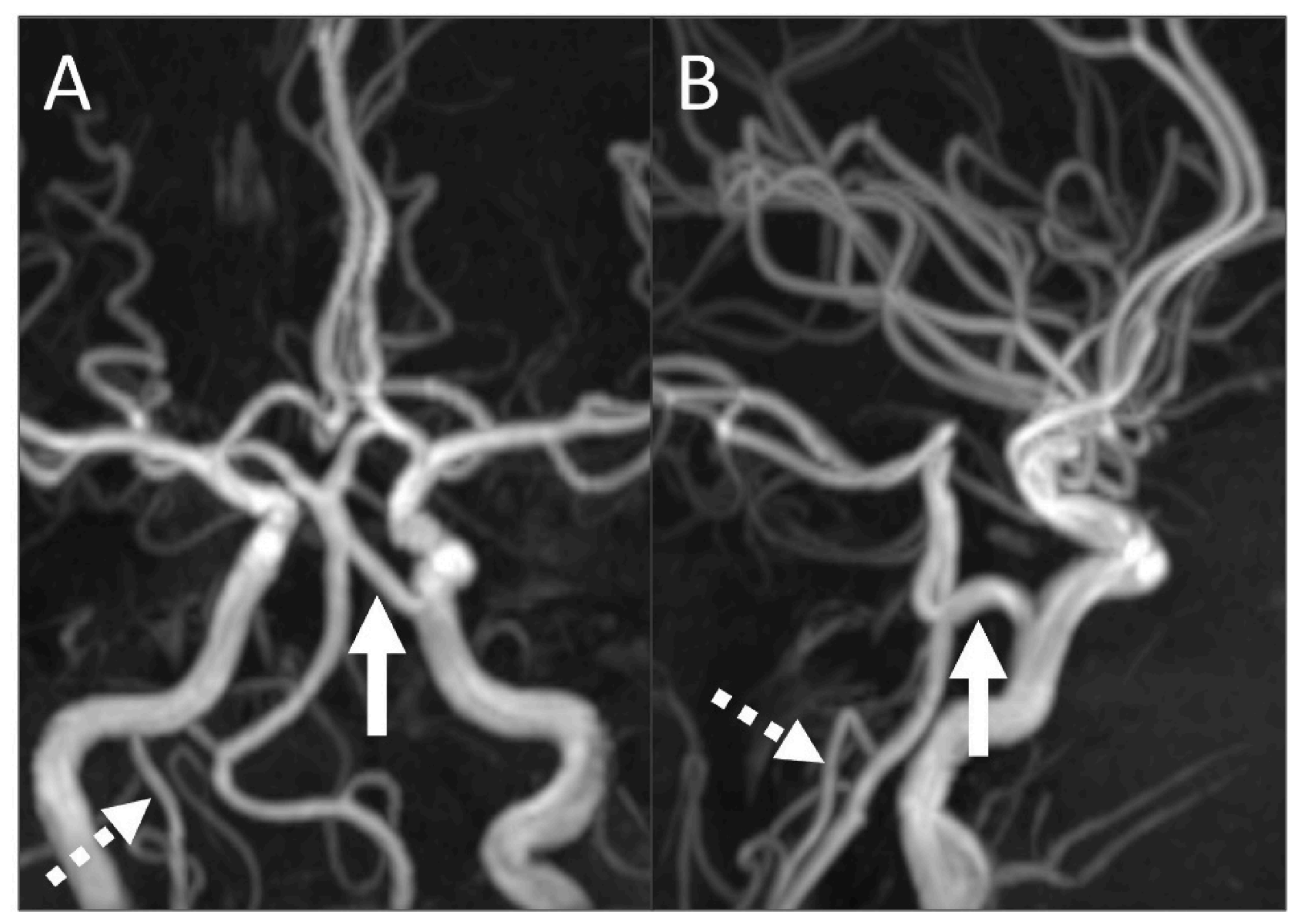
Persistent trigeminal artery. Anteroposterior and lateral projection TOF MRA MIP images (**A**,**B**) demonstrate a left persistent trigeminal artery (white arrows) arising from the posterior bend of the cavernous ICA and joining the distal basilar artery between the origins of the superior cerebellar artery and the anterior inferior cerebellar artery. The proximal basilar artery and the left VA are well developed, while the right VA is slightly hypoplastic (dashed white arrows).

Anatomically, the PTA follows one of two main courses:

− Medial (sphenoidal type): The PTA arises from the cavernous ICA near the region where it leaves the carotid canal and enters the cavernous sinus. It penetrates the sella turcica, courses within its own groove, penetrates the dura near the clivus, and joins the basilar artery between the origins of the superior cerebellar artery (SCA) and anterior inferior cerebellar artery (AICA).
− Lateral (petrosal) type: The PTA leaves the cavernous sinus and courses along the trigeminal root and the lateral aspect of the sella before joining the basilar artery between the origins of the SCA and AICA (Figure 21) [7,52,53].

**Figure 22:**
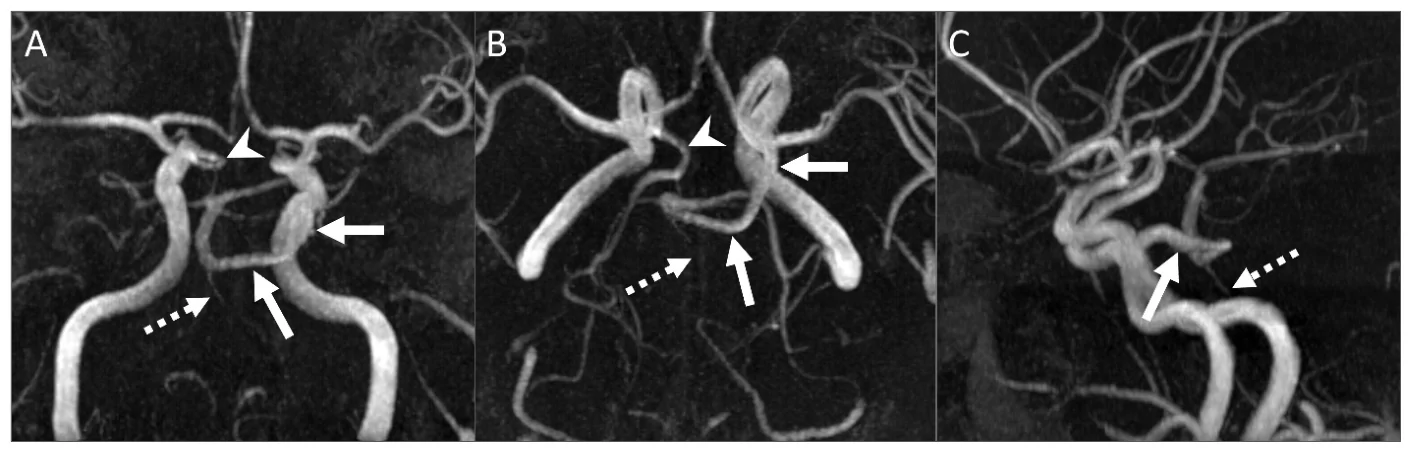
Persistent trigeminal artery in a different patient. TOF MRA MIP images (**A**–**C**) demonstrate a left persistent trigeminal artery (white arrows) arising from the posterior bend of the cavernous ICA and joining the mid-to-distal basilar artery. The proximal basilar artery (dashed white arrows) and the distal VAs are markedly hypoplastic. A right-sided fetal PCA is also present (white arrowhead).

The PTA is generally clinically silent, although it may be associated with trigeminal neuralgia and aneurysm formation at the ICA-PTA junction [54]. Radiologically, the basilar artery proximal to the anastomosis may be small or hypoplastic, and the PTA may coexist with hypoplastic VAs or other persistent embryonic remnants (Figure 22) [7].

#### 5.3.2. Persistent Hypoglossal Artery (PHA)

The PHA is characterized by the persistence of the embryonic hypoglossal artery, which connects the cervical ICA to the basilar artery and traverses the hypoglossal canal [7]. It is usually asymptomatic but may complicate skull base surgery or endovascular procedures. For example, temporary clamping of the PHA during carotid endarterectomy carries a greater ischemic risk in preoperatively unrecognized cases. The PHA may also be associated with hypoglossal nerve palsy, basilar artery aneurysms, or craniocervical junction bone malformations [7,55,56,57].

**Figure 23:**
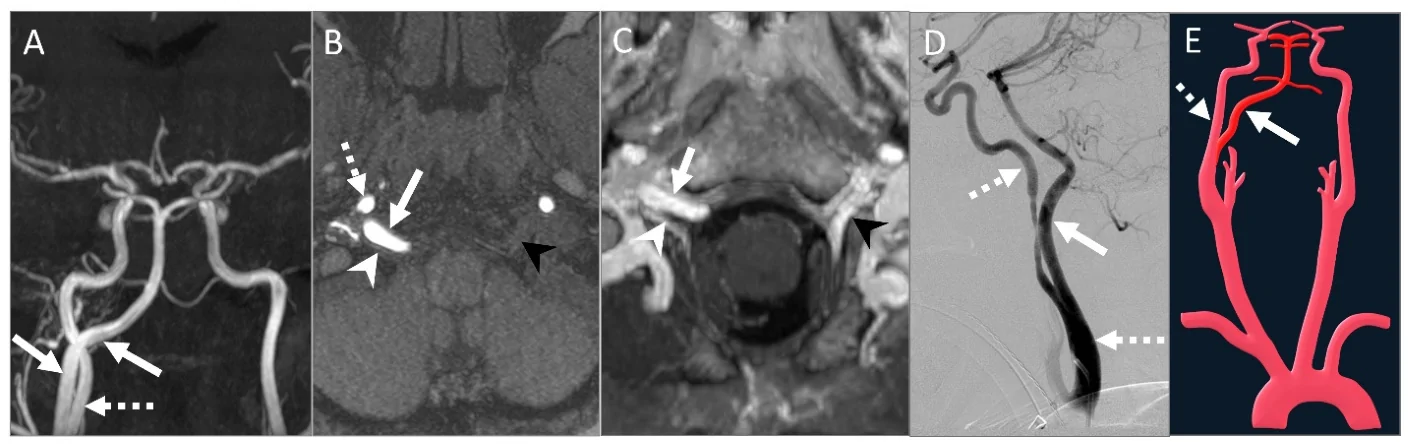
Persistent hypoglossal artery. TOF MRA MIP (**A**), axial TOF MRA source (**B**), post-contrast T1W (**C**), and catheter angiography (**D**) images, alongside an illustration (**E**), demonstrate a persistent hypoglossal artery (white arrows) arising from the right cervical ICA (dashed white arrows) and traversing the right hypoglossal canal (white arrowheads) before joining the vertebrobasilar system. The left hypoglossal canal (black arrowheads) is normal. Multifocal narrowing of the right cervical ICA on conventional angiography is consistent with catheterization-related vasospasm, which subsequently resolved.

On imaging, the PHA appears as a large vessel arising from the cervical ICA between the C1 and C3 levels, entering the hypoglossal canal, and joining the basilar artery. The PComA and the ipsilateral VA are usually absent or hypoplastic (Figure 23). Additionally, enlargement of the hypoglossal canal on bone window CT may serve as a helpful clue to the diagnosis.

#### 5.3.3. Persistent Proatlantal Artery (PPA)

**Figure 24:**
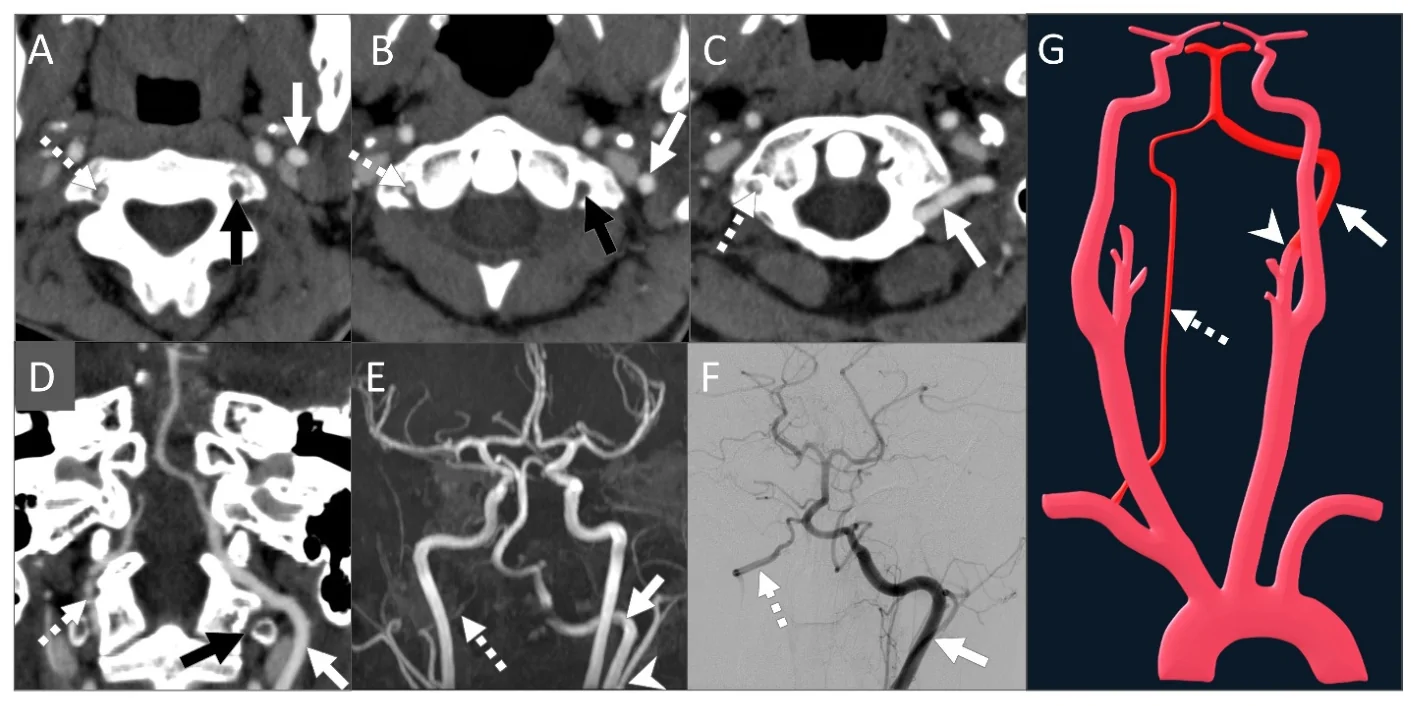
Type 2 persistent proatlantal artery. Axial (**A**–**C**) and coronal (**D**) CTA, TOF MRA MIP (**E**), and left ECA injection catheter angiography (**F**) images, alongside an illustration (**G**), demonstrate a persistent proatlantal artery (white arrows) arising from the left ECA (white arrowheads), coursing dorsally, and joining the left VA at the distal V3 segment in the suboccipital space before entering the foramen magnum. The right VA (dashed white arrows) is hypoplastic, and the left VA V1 through proximal V3 segments are aplastic (black arrows).

The PPA is characterized by the persistence of the primitive proatlantal intersegmental artery, which connects the carotid system to the VA and traverses the foramen magnum. There are two distinct types of PPA:

− Type I: Arises from the cervical ICA and represents the persistence of the true proatlantal artery.
− Type II: Arises from the ECA, often through the occipital artery branch, and corresponds to the persistence of the first cervical intersegmental artery (Figure 24) [7,58].

The PPA is often an incidental finding but may be associated with tinnitus. Notably, this variant anatomy can provide crucial collateral flow in the setting of a VA or subclavian artery occlusion. Recognition of a PPA is critical during craniovertebral junction or skull base surgery, as well as before carotid endarterectomy, ECA embolization, or ligation, because the PPA may serve as the dominant or sole blood supply to the posterior fossa [14,58].

Imaging studies demonstrate a persistent vessel arising from the ICA or ECA that ascends posteromedially to enter the foramen magnum and join the VA. The ipsilateral cervical VA is usually absent or hypoplastic [7,14,15,58].

## 6. Conclusions

Congenital anomalies and variations of the carotid and vertebral arteries result from complex embryologic development and remodeling processes that fundamentally shape vascular anatomy and hemodynamics. These variants, which may involve the origin, course, caliber, branching pattern, or persistence of embryonic connections, are commonly encountered in head and neck imaging studies. Although many of these anomalies are incidental and asymptomatic, some are associated with an increased risk of ischemia, aneurysm formation, cranial neuropathies, or altered collateral circulation.

Modern imaging modalities, including CTA, MRA, and catheter angiography, play complementary roles in defining this complex vascular anatomy and identifying the characteristic features of these variations. Ultimately, familiarity with both the embryologic basis and the distinct imaging appearances of these congenital variants is essential for ensuring accurate interpretation, avoiding diagnostic pitfalls, and safely guiding surgical and endovascular management.

## Data Availability

No new data were created or analyzed in this study. Data sharing is not applicable to this article.

## Abbreviations

The following abbreviations are used in this manuscript:

3D	Three-dimensional
ACA	Anterior cerebral artery
AComA	Anterior communicating artery
AICA	Anterior inferior cerebellar artery
CCA	Common carotid artery
CISA	Cervical intersegmental artery
COW	Circle of Willis
CTA	Computed tomography angiography
DSA	Digital subtraction angiography
ECA	External carotid artery
ICA	Internal carotid artery
LNA	Longitudinal neural artery
MCA	Middle cerebral artery
MIP	Maximum intensity projection
MMA	Middle meningeal artery
MRA	Magnetic resonance angiography
PComA	Posterior communicating artery
PHA	Persistent hypoglossal artery
PPA	Persistent proatlantal artery
PSA	Persistent stapedial artery
PTA	Persistent trigeminal artery
PCD	Photon-counting detector
PICA	Posterior inferior cerebellar artery
SCA	Superior cerebellar artery
TOF	Time of flight
VA	Vertebral artery
VR	Volume-rendered
VWI	Vessel wall imaging
